# Supplementary material for: GDF15 Provides an Endocrine Signal of Nutritional Stress in Mice and Humans
Source: Cell Metab. 2019 Mar 5;29(3):707–718.e8. doi: 10.1016/j.cmet.2018.12.016 (PMC6408327; doi:10.1016/j.cmet.2018.12.016)
Supplement: Document S1. Figures S1–S4 [file mmc1.pdf]

Cell Metabolism, Volume *29*

Supplemental Information

GDF15 Provides an Endocrine Signal of Nutritional Stress in Mice and Humans

Satish Patel, Anna Alvarez-Guaita, Audrey Melvin, Debra Rimmington, Alessia Dattilo, Emily L. Miedzybrodzka, Irene Cimino, Anne-Catherine Maurin, Geoffrey P. Roberts, Claire L. Meek, Samuel Virtue, Lauren M. Sparks, Stephanie A. Parsons, Leanne M. Redman, George A. Bray, Alice P. Liou, Rachel M. Woods, Sion A. Parry, Per B. Jeppesen, Anders J. Kolnes, Heather P. Harding, David Ron, Antonio Vidal-Puig, Frank Reimann, Fiona M. Gribble, Carl J. Hulston, I. Sadaf Farooqi, Pierre Fafournoux, Steven R. Smith, Jorgen Jensen, Danna Breen, Zhidan Wu, Bei B. Zhang, Anthony P. Coll, David B. Savage, and Stephen O'Rahilly

Supplemental figures

GDF 15 provides an endocrine signal of nutritional stress in mice and humans

Satish Patel, Anna Alvarez-Guaita, Audrey Melvin, Debra Rimmington, Alessia Dattilo, Emily L. Miedzybrodzka, Irene Cimino, Anne-Catherine Maurin, Geoffrey P. Roberts, Claire L. Meek, Samuel Virtue, Lauren M. Sparks, Stephanie A. Parsons, Leanne M. Redman, George A. Bray, Alice P. Liou, Rachel M. Woods, Sion A. Parry, Per B. Jeppesen, Anders J. Kolnes , Heather P. Harding, David Ron, Antonio Vidal-Puig, Frank Reimann, Fiona M. Gribble, Carl J. Hulston, I. Sadaf Farooqi, Pierre Fafournoux[3], Steven R. Smith , Jorgen Jensen, Danna Breen, Zhidan Wu, Bei B. Zhang, Anthony P. Coll, David B. Savage, Stephen O'Rahilly

Figure S1

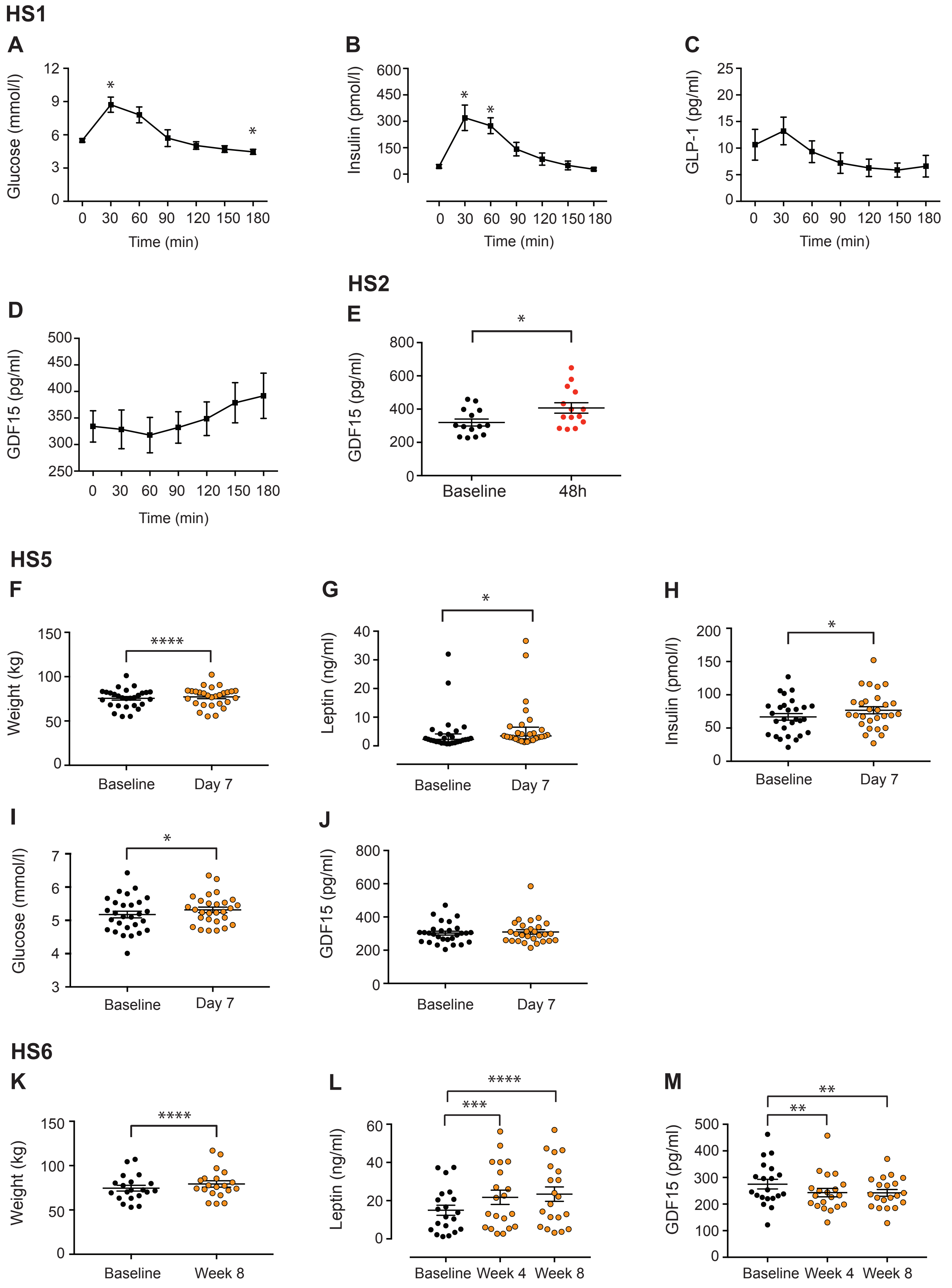

HS1
A
Glucose (mmol/l)
Time (min)
B
Insulin (pmol/l)
Time (min)
C
GLP-1 (pg/ml)
Time (min)
D
GDF15 (pg/ml)
Time (min)
HS2
E
GDF15 (pg/ml)
Baseline
48h
HS5
F
Weight (kg)
Baseline
Day 7
G
Leptin (ng/ml)
Baseline
Day 7
H
Insulin (pmol/l)
Baseline
Day 7
I
Glucose (mmol/l)
Baseline
Day 7
J
GDF15 (pg/ml)
Baseline
Day 7
HS6
K
Weight (kg)
Baseline
Week 8
L
Leptin (ng/ml)
Baseline
Week 4
Week 8
M
GDF15 (pg/ml)
Baseline
Week 4
Week 8

Supplemental Figure Legends

Figure S1 (related to Figure 1). **GDF15 levels in response to a meal or imposed caloric deficit in mice and humans.**

HS1-Human Study 1 (A-D): Plasma (A) glucose, (B) insulin, (C) GLP-1 and (D) GDF15 circulating levels in six healthy volunteers given an 50 g oral glucose tolerance test following an overnight fast. Blood samples were taken at serial intervals over the 180 min duration of the study. Data is expressed as mean ± SEM. * p <0.05 comparing to Time 0 min by a One-way ANOVA with Bonferroni post-test.

HS2- Human study 2 (E) GDF15 levels of 14 healthy male volunteers undergoing 48 h of caloric restriction to 10 % of estimated energy requirement per day. Data is expressed as mean (SEM) and compared using a paired t-test, * p <0.05.

HS5-Human study 5 (F-J): (F) Body weight, (G) leptin, (H) insulin, (I) glucose and (J) GDF15 levels before and after 7 days of overfeeding (48 ± 1 % greater than estimated daily requirements) in non-obese healthy human volunteers. Data is from 28 adult participants and expressed as mean ± SEM compared using a paired Two tailed Student t-test, with leptin expressed as median (IQR) compared using a Wilcoxon signed rank test, *p <0.05, **** p <0.0001.

HS6-Human study 6 (K-M)**:** (K) Body weight (L) leptin and (M) GDF15 following four or eight weeks of overfeeding (additional 40 % weight maintenance energy requirements) in 20 volunteers. Data is expressed as mean ± SEM. p < 0.01, ****p < 0.0001 by a One-way ANOVA with Bonferroni multiple comparison post-test **

Figure S2

MS2

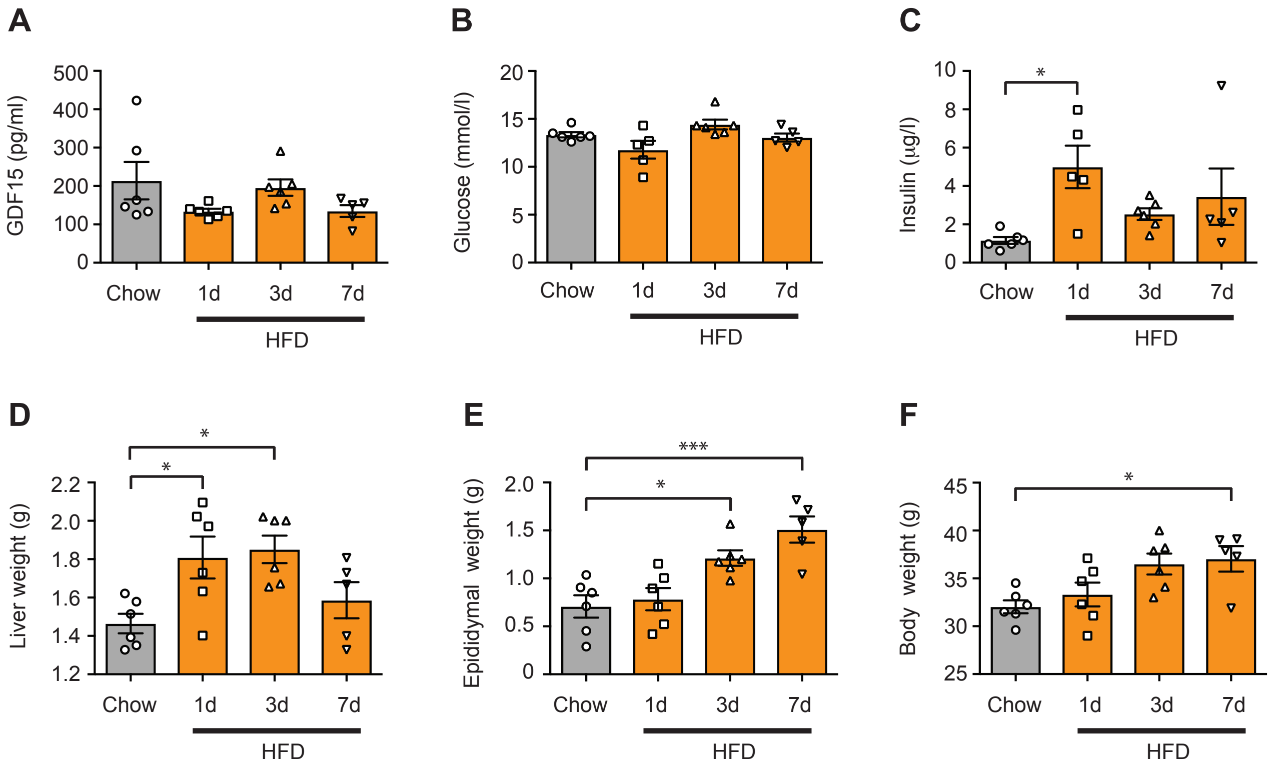

Figure S2 (related to Figure 2): **GDF15 is unaffected by acute HFD (high-fat diet) feeding in mice.**

MS2-Mouse study 2: Plasma (A) GDF15, (B) blood glucose and (C) plasma insulin concentrations from 17-18 week-old male mice that were either fed a chow diet or subjected to a 45% HFD for 1-7 days (d). (D) Liver, (E) epididymal adipose tissue and (F) body weight from each diet group. Data is expressed as mean ± SEM (n=6 mice per group, except 7d, n=5). * $p<0.05$, ***$p < 0.001$ by One way ANOVA with Bonferroni multiple comparison post test.

Figure S3

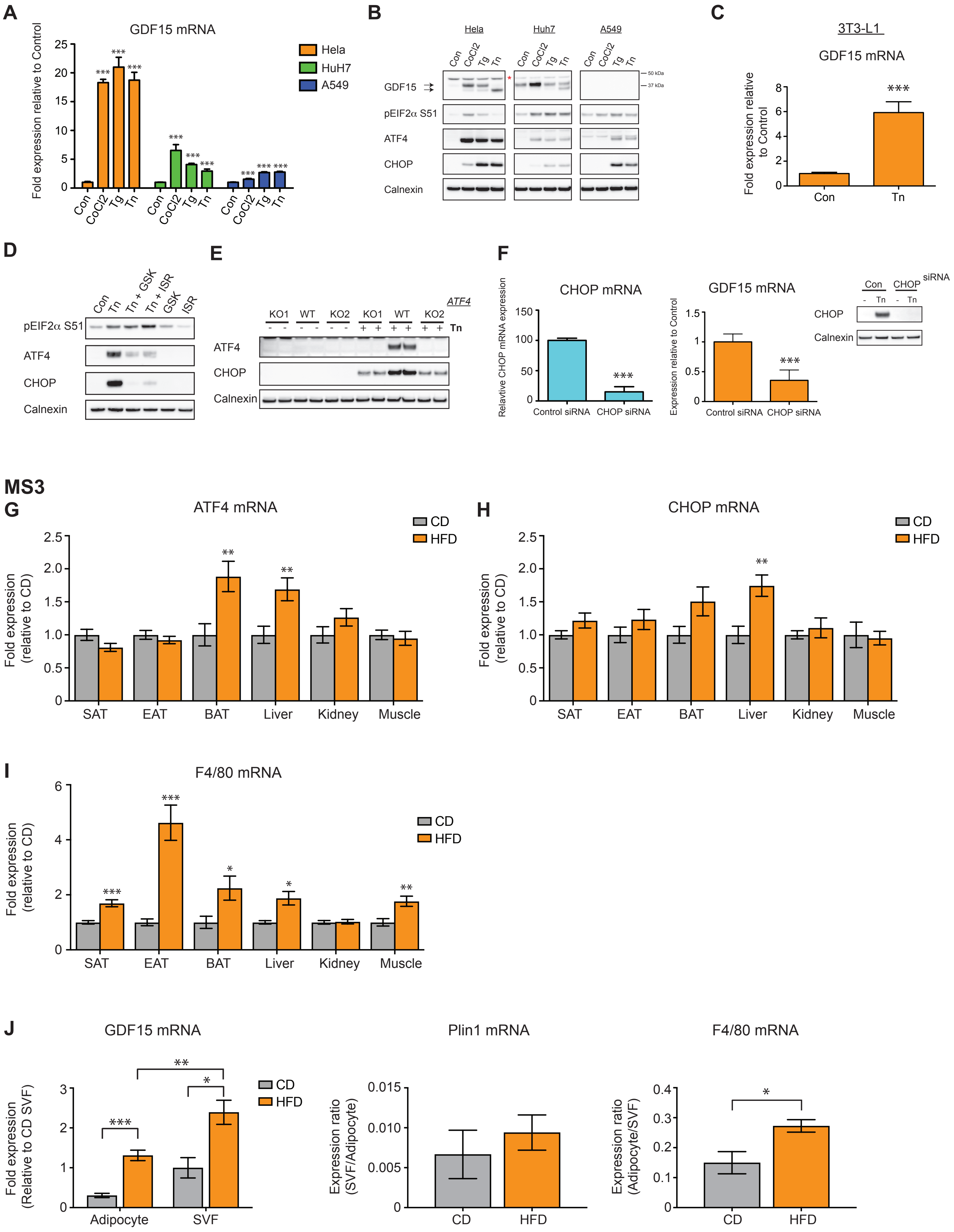

A
GDF15 mRNA
Fold expression relative to Control
Hela
HuH7
A549
Con
CoCl2
Tg
Tn
B
Hela
Huh7
A549
GDF15
pEIF2α S51
ATF4
CHOP
Calnexin
50 kDa
37 kDa
C
3T3-L1
GDF15 mRNA
Fold expression relative to Control
Con
Tn
D
Con
Tn
Tn + GSK
Tn + ISR
GSK
ISR
pEIF2α S51
ATF4
CHOP
Calnexin
E
ATF4
KO1
WT
KO2
Tn
ATF4
CHOP
Calnexin
F
CHOP mRNA
Relative CHOP mRNA expression
Control siRNA
CHOP siRNA
GDF15 mRNA
Expression relative to Control
Con
CHOP
siRNA
CHOP
Calnexin
MS3
G
ATF4 mRNA
Fold expression (relative to CD)
CD
HFD
SAT
EAT
BAT
Liver
Kidney
Muscle
H
CHOP mRNA
Fold expression (relative to CD)
I
F4/80 mRNA
Fold expression (relative to CD)
J
GDF15 mRNA
Fold expression (Relative to CD SVF)
Adipocyte
SVF
Plin1 mRNA
Expression ratio (SVF/Adipocyte)
F4/80 mRNA
Expression ratio (Adipocyte/SVF)

Figure S3 (related to Figure 3). GDF15 is regulated by the cellular integrated stress (ISR) response pathway. (A) GDF15 mRNA expression and (B) immunoblot analysis from cell lysates for ISR components in Hela, HuH7 and A549 cells treated with cobalt chloride (CoCl2, 625 uM), thapsigargin (Tg, 1 μM), tunicamycin (Tn 5 μg/ml) or L-Histidinol (His, 1 mM) for 6 h. Note for (B), the arrows denote GDF15 protein and that Tn treatment causes a mobility shift that we hypothesize is due to an impairment of GDF15 glycosylation. Red asterisks indicates a non-specific band. Whilst GDF15 mRNA was induced in (A), there was no detectable GDF15 protein in A549 cells in cell lysates. (C) GDF15 mRNA expression in 3T3-L1 preadipocytes treated with Tn (5 μg/ml) for 6 h. (D) Immunoblot analysis from cell lysates for ISR components in WT MEFs treated with TN in the presence or absence of the PERK inhibitor GSK2606414 (GSK, 200 nM) or eIF2a inhibitor ISRIB (ISR, 100 nM) or (E) in Tn-treated WT or ATF4 KO MEFs. GDF15 mRNA is presented as fold expression relative to its respective control treatment for each cell type (set at 1), normalised to HPRT gene expression in MEFs and 3T3-L1 and GAPDH in human cells. Data is expressed as mean ± SD from three independent experiments. Blots shown are a representative of three independent experiments with Calnexin used as a loading control. (F) CHOP or GDF15 mRNA expression in control siRNA and CHOP siRNA transfected WT MEFs, right hand panel, immunoblot analysis from cell lysates showing the effectiveness of CHOP siRNA on Tn-induced (5 μg/ml – 6 h) CHOP protein expression. mRNA data is mean ± SD from three independent experiments with control treated cells set as 100. ***p < 0.001 by Two tailed Student T-Test. Blot shown is a representative of three independent experiments with Calnexin used as a loading control.

GDF15 upregulation in high-fat fed mice is associated with induction of ISR (integrated stress response) pathways. MS3-Mouse Study 3: (A) ATF4 (B) CHOP and (C) F4/80 mRNA expression in subcutaneous - (SAT), epididymal - (EAT) and brown (BAT) adipose tissue, liver, soleus muscle and kidney isolated from C57Bl/6J male mice fed a chow - (CD) or high-fat diet (HFD) for 18 weeks (n = 6-8 mice/group). mRNA is presented as fold expression (mean ± SEM) relative to CD (set at 1) and normalised to the geometric mean of B2M/36b4 gene expression. * p <0.05, ** p < 0.01, ***p < 0.001 by Two tailed Student T-Test. (D) GDF15, Plin1 and F4/80 mRNA expression in adipocyte and stromo- vascular fractions (SVF) from 18 weeks CD or HFD epididymal adipose tissue. For GDF15, mRNA data is presented as fold-expression relative to chow fed SVF, whereas for Plin1 and F4/80, is presented as ratio between the two fractions, with all data normalised to geometric mean of 36b4/HPRT. Data is expressed as mean ± SEM and analysed by Two way ANOVA with Bonferroni multiple comparison post test for GDF15 and Two tailed Student T-Test for Plin1 and F4/80 * p <0.05, ** p < 0.01, ***p < 0.001.

Figure S4

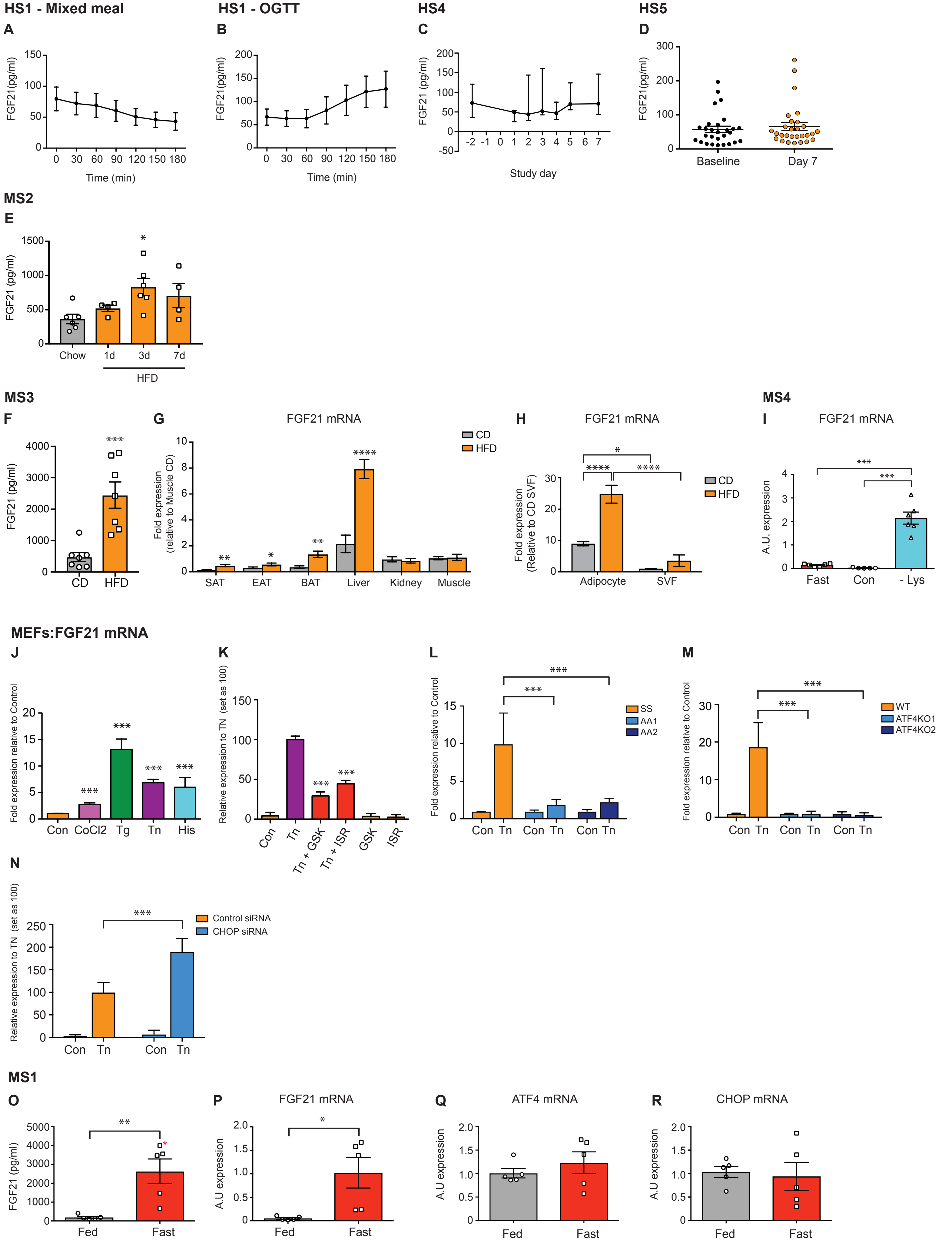
HS1 - Mixed meal
A
FGF21(pg/ml)
Time (min)
HS1 - OGTT
B
FGF21(pg/ml)
Time (min)
HS4
C
FGF21 (pg/ml)
Study day
HS5
D
FGF21(pg/ml)
Baseline
Day 7
MS2
E
FGF21 (pg/ml)
Chow
1d
3d
7d
HFD
MS3
F
FGF21 (pg/ml)
CD
HFD
G
FGF21 mRNA
Fold expression (relative to Muscle CD)
CD
HFD
SAT
EAT
BAT
Liver
Kidney
Muscle
H
FGF21 mRNA
Fold expression (Relative to CD SVF)
CD
HFD
Adipocyte
SVF
MS4
I
FGF21 mRNA
A.U. expression
Fast
Con
- Lys
MEFs:FGF21 mRNA
J
Fold expression relative to Control
Con
CoCl2
Tg
Tn
His
K
Relative expression to TN (set as 100)
Con
Tn
Tn + GSK
Tn + ISR
GSK
ISR
L
Fold expression relative to Control
SS
AA1
AA2
Con
Tn
M
Fold expression relative to Control
WT
ATF4KO1
ATF4KO2
Con
Tn
N
Relative expression to TN (set as 100)
Control siRNA
CHOP siRNA
Con
Tn
MS1
O
FGF21 (pg/ml)
Fed
Fast
P
FGF21 mRNA
A.U expression
Fed
Fast
Q
ATF4 mRNA
A.U expression
Fed
Fast
R
CHOP mRNA
A.U expression
Fed
Fast

Figure S4 (related to Figures 1-4). FGF21 regulation in response to nutritional challenges in mice and humans.

HS1-5 – Human Studies 1-5. (A-C) Circulatory levels of FGF21 in volunteers that participated in meal or imposed caloric deficit or (D) overfeeding studies. MS2-4- Mouse Studies 2-4. Plasma FGF21 and tissue mRNA expression in mice subjected to (E) short-term or (F-H) Long-term high fat or (I) lysine deficient diet. (J-N) FGF21 mRNA expression and its regulation by the cellular intergrated stress response pathways in MEFs . MS1- Mouse study 1. (O) Plasma FGF21 and (P-R) hepatic FGF21, ATF4 and CHOP mRNA expression in mice subjected to fasting. The experimental details and the statistical analysis for FGF21 are identical to those conducted for GDF15 (see main and supplemental figures).
