# Supplementary material for: GDF15 Provides an Endocrine Signal of Nutritional Stress in Mice and Humans
Source: Cell Metab. 2019 Mar 5;29(3):707–718.e8. doi: 10.1016/j.cmet.2018.12.016 (PMC6408327; doi:10.1016/j.cmet.2018.12.016)
Supplement: Document S2. Article plus Supplemental Information [file mmc2.pdf]

Short Article

Cell Metabolism

GDF15 Provides an Endocrine Signal of Nutritional Stress in Mice and Humans

Graphical Abstract

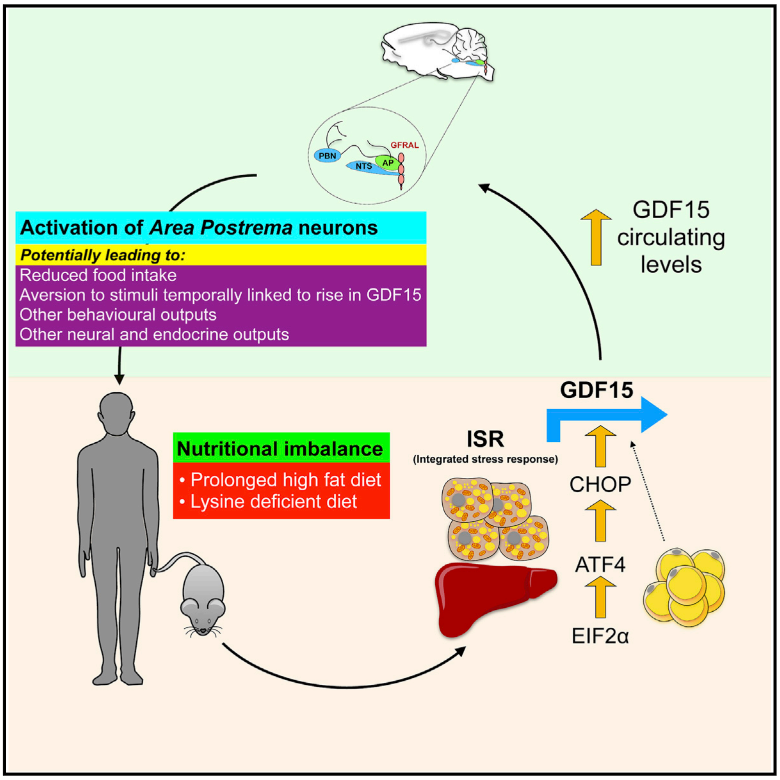

Highlights

- Dietary changes influencing adipose/gut-derived hormones do not alter GDF15 levels
- Chronic high-fat or acute lysine-deficient diet exposure increases GDF15 levels
- GDF15 administration triggers conditioned taste aversion in mice
- GDF15 is a stress-induced hormone that may mediate an aversive dietary response

Authors

Satish Patel, Anna Alvarez-Guaita, Audrey Melvin, ..., Anthony P. Coll, David B. Savage, Stephen O'Rahilly

Correspondence

dbs23@medschl.cam.ac.uk (D.B.S.), so104@medschl.cam.ac.uk (S.O.)

In Brief

Patel et al. show that whereas short-term overfeeding or fasting does not change GDF15 levels substantially, prolonged high-fat feeding and lysine-deficient diets activate the integrated stress response and increase GDF15 levels. GDF15 administration triggers conditioned taste aversion in mice, suggesting that GDF15 might induce an aversive response to nutritional stress.

Patel et al., 2019, Cell Metabolism *29*, 707–718
March 5, 2019 © 2018 The Author(s). Published by Elsevier Inc.
https://doi.org/10.1016/j.cmet.2018.12.016

CellPress

GDF15 Provides an Endocrine Signal of Nutritional Stress in Mice and Humans

Satish Patel,[1,12] Anna Alvarez-Guaita,[1,12] Audrey Melvin,[1,12] Debra Rimmington,[1] Alessia Dattilo,[1] Emily L. Miedzybrodzka,[1] Irene Cimino,[1] Anne-Catherine Maurin,[3] Geoffrey P. Roberts,[1] Claire L. Meek,[1] Samuel Virtue,[1] Lauren M. Sparks,[6] Stephanie A. Parsons,[6] Leanne M. Redman,[7] George A. Bray,[7] Alice P. Liou,[4] Rachel M. Woods,[5] Sion A. Parry,[5,11] Per B. Jeppesen,[8] Anders J. Kolnes,[9] Heather P. Harding,[1,2] David Ron,[1,2] Antonio Vidal-Puig,[1] Frank Reimann,[1] Fiona M. Gribble,[1] Carl J. Hulston,[5] I. Sadaf Farooqi,[1] Pierre Fafournoux,[3] Steven R. Smith,[6] Jorgen Jensen,[10] Danna Breen,[4] Zhidan Wu,[4] Bei B. Zhang,[4] Anthony P. Coll,[1,13] David B. Savage,[1,13,14,*] and Stephen O'Rahilly[1,13,*]

[1]Metabolic Research Laboratories, Wellcome Trust-Medical Research Council Institute of Metabolic Science, University of Cambridge, Cambridge CB2 0QQ, UK
[2]Cambridge Institute for Medical Research, Cambridge University, Cambridge CB2 0XY, UK
[3]INRA, Unité de Nutrition Humaine, Université Clermont Auvergne, 63000 Clermont-Ferrand, France
[4]Internal Medicine Research Unit, Pfizer Global R&D, 1 Portland Street, Cambridge, MA, USA
[5]School of Sport, Exercise and Health Sciences, Loughborough University, Loughborough, Leicestershire LE11 3TU, UK
[6]Translational Research Institute for Metabolism and Diabetes, Florida Hospital, Orlando, FL, USA
[7]Pennington Biomedical Research Center, Baton Rouge, LA, USA
[8]Department of Clinical Medicine, Aarhus University Hospital, Aarhus University, Aarhus, Denmark
[9]Section of Specialized Endocrinology, Department of Endocrinology, Oslo University Hospital, Rikshospitalet, Oslo, Norway
[10]Department of Physical Performance, Norwegian School of Sport Sciences, Oslo, Norway
[11]Present address: Metabolic Research Group, Oxford Centre for Diabetes, Endocrinology and Metabolism, Radcliffe Department of Medicine, University of Oxford, Oxford OX3 7LE, UK
[12]These authors contributed equally
[13]Senior author
[14]Lead Contact
*Correspondence: dbs23@medschl.cam.ac.uk (D.B.S.), so104@medschl.cam.ac.uk (S.O.)
https://doi.org/10.1016/j.cmet.2018.12.016

SUMMARY

GDF15 is an established biomarker of cellular stress. The fact that it signals via a specific hindbrain receptor, GFRAL, and that mice lacking GDF15 manifest diet-induced obesity suggest that GDF15 may play a physiological role in energy balance. We performed experiments in humans, mice, and cells to determine if and how nutritional perturbations modify GDF15 expression. Circulating GDF15 levels manifest very modest changes in response to moderate caloric surpluses or deficits in mice or humans, differentiating it from classical intestinally derived satiety hormones and leptin. However, GDF15 levels do increase following sustained high-fat feeding or dietary amino acid imbalance in mice. We demonstrate that GDF15 expression is regulated by the integrated stress response and is induced in selected tissues in mice in these settings. Finally, we show that pharmacological GDF15 administration to mice can trigger conditioned taste aversion, suggesting that GDF15 may induce an aversive response to nutritional stress.

INTRODUCTION

GDF15 (growth differentiation factor 15; also known as macrophage inhibitory cytokine-1 [MIC-1], NAG1, PLAB, and PDF) is a stress-induced cytokine and an atypical member of the transforming growth factor beta superfamily (Tsai et al., 2016). Bootcov et al. originally characterized it as a dimeric protein secreted by activated macrophages (Bootcov et al., 1997). In healthy animals, it is predominantly expressed in the liver, lung, and kidney and, at least in humans, in large amounts in the placenta (Böttner et al., 1999; Ding et al., 2009; Fairlie et al., 1999; Lawton et al., 1997; Marjono et al., 2003; Yokoyama-Kobayashi et al., 1997). It circulates at high levels in humans (Brown et al., 2003; Ho et al., 2012; Kempf et al., 2007; Mullican and Rangwala, 2018) and serum levels are known to increase with age, smoking, intense exercise, cancer, and a range of other disease states (reviewed in Corre et al., 2013; Kleinert et al., 2018; Unsicker et al., 2013). It appears that almost any cell or tissue can express GDF15 in response to various forms of stress (Appierto et al., 2009; Chung et al., 2017; Hsiao et al., 2000; Park et al., 2012; Yang et al., 2010). The measurement of circulating concentrations of GDF15 is beginning to enter clinical practice as a diagnostic biomarker in mitochondrial disease and as a prognostic marker in conditions such as heart failure and certain cancers (Fujita et al., 2016; Wang et al., 2013; Wollert et al., 2017).

Johnen et al. first reported that mice bearing tumors engineered to overexpress GDF15 lost weight dramatically (Johnen et al., 2007). This could also be reproduced by injection of recombinant GDF15 and prevented by a neutralizing GDF15 antibody. Transgenic GDF15-expressing mice similarly lost weight secondary to reduced food intake (Chrysovergis et al., 2014; Macia et al., 2012). Conversely, GDF15 null mice were

 © 2018 The Author(s). Published by Elsevier Inc.
This is an open access article under the CC BY license (http://creativecommons.org/licenses/by/4.0/).

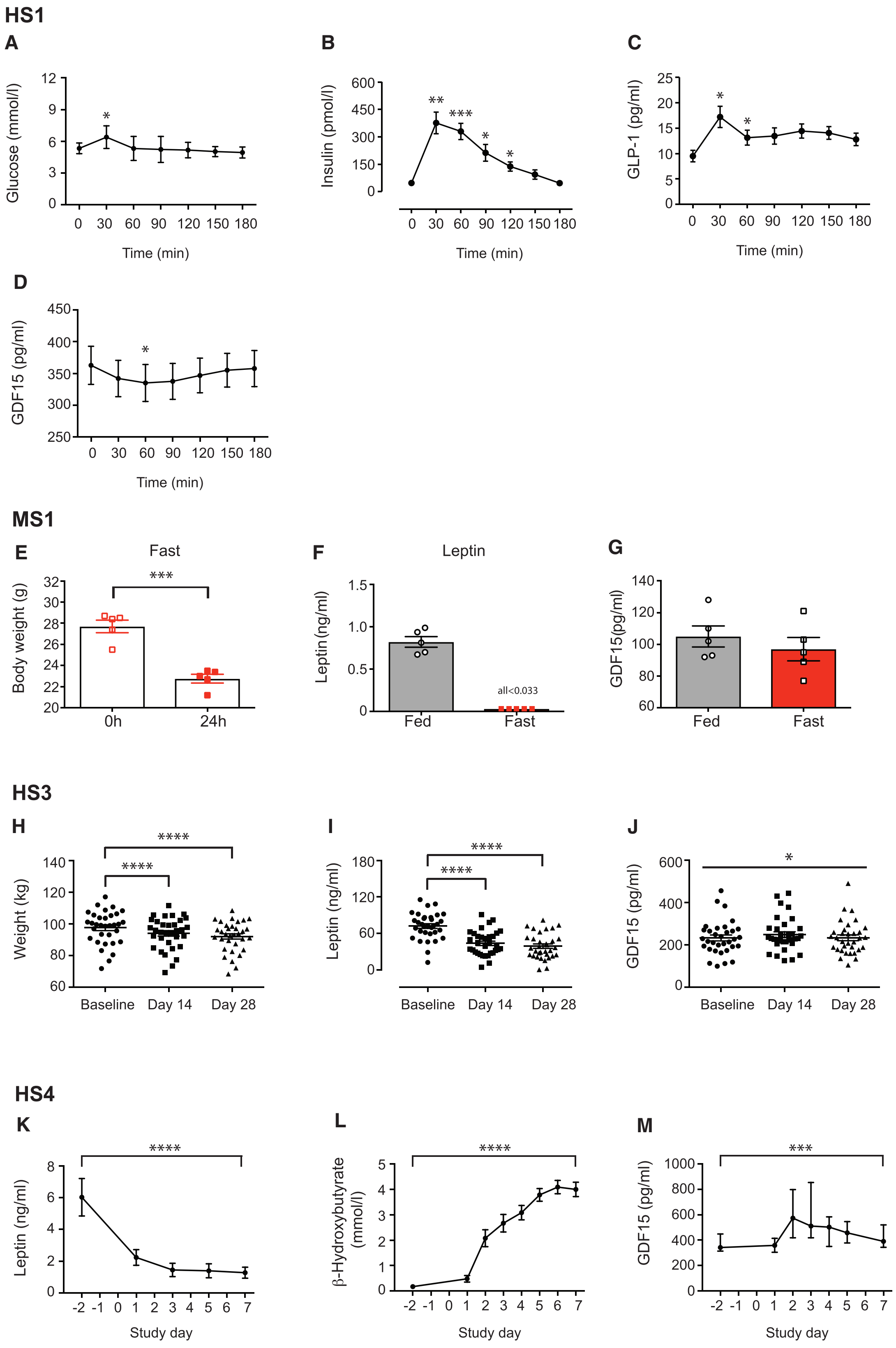

(legend on next page)

reported to be slightly heavier (6%–10%) than their wild-type littermates (Tsai et al., 2013) on a chow diet (CD), a difference that becomes more striking on a high-fat diet (HFD) (Tran et al., 2018). GDF15 injection induced cFos activation in selected regions of the brainstem, particularly the nucleus tractus solitarius (NTS) and area prostrema (AP). Selective lesioning of these hindbrain regions rendered mice unresponsive to the anorexigenic actions of GDF15 (Tsai et al., 2014). Recently, it has been demonstrated that these effects of GDF15 are mediated via a receptor composed of a heterodimer of Ret and a member of the GDNF receptor alpha (GFRa) family, known as GFRa-like or GFRAL (Emmerson et al., 2017; Hsu et al., 2017; Mullican et al., 2017; Yang et al., 2017). Notably, GFRAL expression appears to be strictly confined to the AP and NTS. In addition to reporting the structure of GFRAL, these papers also showed that GFRAL knockout (KO) mice are resistant to the anorectic effects of exogenous injected GDF15 and to endogenously secreted GDF15 levels induced by cisplatin chemotherapy (Hsu et al., 2017), clearly establishing the GDF15-GFRAL axis as critical to stress pathway-induced weight loss. Interestingly, two groups also noted that whereas body weight is similar to that of wild-type littermates in chow-fed GFRAL null mice, GFRAL null animals gain more weight on an HFD (Hsu et al., 2017; Mullican and Rangwala, 2018), whereas the other groups reported similar body weights in GFRAL null mice on an HFD (Emmerson et al., 2017; Yang et al., 2017). It is unknown whether circulating levels of GDF15 rise in response to sustained overfeeding and, if this occurs, what tisues are responsible.

Here, we explore the relationship between GDF15 production and nutritional state and find that, in contrast to enteroendocrine hormones or leptin, GDF15 levels are not influenced by meals, by the imposition of periods of caloric deficit or caloric excess of moderate intensity and duration in mice or humans. However, GDF15 levels do increase significantly when mice are exposed to chronic high-fat feeding. We then characterize in detail the elements of the cellular integrated stress response (ISR) that are involved in the regulation of GDF15 expression and demonstrate activation of the ISR in selected tissues of high-fat-fed mice. We also show that another severe nutritional perturbation, namely provision of a lysine-deficient diet to mice, activates the ISR and increases GDF15 levels. Finally, we provide the first evidence that GDF15 generates an aversive signal through the demonstration of conditioned taste aversion (CTA) in mice.

RESULTS

GDF15 Levels Are Unaffected by Meals or Glucose Ingestion

It is well established in humans that hormones derived from enteroendocrine cells respond to acute changes in nutritional state and play a key role in regulating energy homeostasis. To determine if GDF15 responds in a similar way, we studied the response of humans to established stimuli of the enteroendocrine system.

In Human Study 1, fasting (overnight) healthy volunteers received a mixed macronutrient liquid drink (Figures 1A–1D) or 50 g anhydrous glucose (Figures S1A–S1D) followed by 30-min blood sampling for 180 min. In both studies, glucose peaked at 30 min (6.60 ± 0.26 mmol/L and 8.71 ± 0.16 mmol/L, respectively), with a lower blood glucose in the mixed macronutrient load reflecting the lower sugar content (22 g) in the test drink. In parallel with the glucose peak, early increases in both insulin and GLP-1 levels were observed, whereas circulating GDF15 levels briefly (at 60 min time point) fell after the mixed meal and were unchanged following glucose ingestion, as reported previously (Tsai et al., 2015).

GDF15 Levels in Response to an Imposed Caloric Deficit

A fall in the adipose-derived hormone leptin represents an important peripheral signal of nutritional deprivation, serving to induce hyperphagia and suppress selected neuroendocrine hormone axes. To address the question of whether GDF15 mirrored the behavior of leptin to changes in nutritional state, we evaluated the response of circulating GDF15 to fasting and caloric restriction in mice and humans.

First, we examined hormone responses to a 24 h fast in mice (Mouse Study 1). Despite a 17.8% loss in body weight and a marked fall in leptin levels, circulating levels of GDF15 were unchanged (Figures 1E–1G).

Next, circulating GDF15 levels were measured in three independent studies in humans subjected to caloric restriction of varying intensity and duration. In Human Study 2, GDF15 concentrations increased from 319.4 ± 21.27 pg/mL to 406.8 ± 31.24 pg/mL in lean healthy volunteers calorie restricted for 2 days (10% of estimated daily energy requirements) (Figure S1E).

In Human Study 3, a cohort of obese participants consumed a low-calorie meal replacement diet (~1,000 kcal/day) for 28 days. This resulted in a significant reduction in body weight

Figure 1. GDF15 Levels in Response to a Meal or Imposed Caloric Deficit in Mice and Humans

(A–D) Human Study 1 (HS1): (A) plasma glucose, (B) insulin, (C) GLP-1, and (D) GDF15 circulating levels in six healthy volunteers given an oral mixed macronutrient liquid meal following an overnight fast. Blood samples were taken over the 180 min duration of the study. Data are expressed as mean ± SEM. *$p < 0.05$, **$p < 0.01$, ***$p < 0.001$ compared to time 0 min by one-way ANOVA with Bonferroni post-test.

(E–G) Mouse Study 1 (MS1): (E) body weight of mice before and after a 24 h fasting challenge (n = 5 mice). (F) Leptin and (G) GDF15 serum concentrations in 11- to 12-week-old male mice either in the fed state or after a 24 h fast. Data are expressed as mean ± SEM (n = 5 mice per group). ***$p < 0.001$ by two-tailed Student's t test. Note all fasted leptin values were under the detection limit (0.033 ng/mL).

(H–J) Human Study 3 (HS3): (H) body weight, (I) leptin, and (J) GDF15 levels in a cohort of overweight and obese participants subjected to caloric restriction (~1,000 kcal/day) for a period of 28 days. Data are from 33 participants, expressed as mean ± SEM. *$p < 0.05$, ****$p < 0.0001$ by a one-way ANOVA with Bonferroni multiple comparison post-test (for body weight and leptin).

(K–M) Human Study 4 (HS4): (K) leptin, (L) β-hydroxybutyrate, and (M) GDF15 levels in human volunteers subjected to a 7-day fast (0 kcal per day). Data are from 13 participants, expressed as mean ± SEM and analyzed by a one-way ANOVA. In the case of GDF15, values are expressed as median (interquartile range). ***$p < 0.001$, ****$p < 0.0001$ by Kruskal-Wallis test. See also Figures S1 and S4.

(−5.55 ± 2.05 kg from baseline, $p < 0.0001$; Figure 1H) and leptin levels (Figure 1I), whereas there was a small, statistically significant increase in GDF15 levels (Figure 1J).

In Human Study 4, a group of lean healthy participants underwent 7 days of total calorie deprivation. As expected, circulating leptin levels fell precipitously from 6.03 ± 1.18 ng/mL to 2.24 ± 0.49 ng/mL at 24 h (Figure 1K) and a marked ketogenic response (β-hydroxubutyrate) was observed in response to the fast (Figure 1L). Meanwhile, circulating GDF15 levels increased, peaking at 48 h of caloric restriction (from 371.4 ± 94.2 pg/mL at baseline to 670.2 ± 349.2 pg/mL, $p = 0.003$). Interestingly, despite continuation of the calorie deprivation, GDF15 levels gradually returned toward baseline levels, although they remained higher than starting values (441.2 ± 151.3 pg/mL) (Figure 1M).

From these studies in both mice and humans, it is clear that GDF15 does not exhibit a leptin-like response to caloric restriction. Rather, a small increase in GDF15 levels is observed under conditions of severe nutritional deprivation.

GDF15 in Response to Short-Term Hypercaloric Loads

In contrast to caloric deprivation, states of nutritional excess and weight gain are associated with physiological increases in leptin. We studied the effect of short-term hyper-caloric interventions on non-obese volunteers to determine if they had a similar effect on GDF15.

In Human Study 5, we assessed changes in GDF15 in healthy volunteers in response to short-term high-fat overfeeding. After 7 days of the intervention, a significant increase in body weight was observed (1.64 ± 1.07 kg, $p < 0.0001$) compared to baseline (Figure S1F), accompanied by a significant increase in leptin levels (Figure S1G). Despite the observed increases in fasting insulin and glucose levels (Figures S1H and S1I), there was no significant change in median (IQR) GDF15 levels (302.0 [256.0–318.0] pg/mL [baseline] versus 295.0 [258.0–343.5] pg/mL [after overfeeding]) (Figure S1J).

Human Study 6 examined the effect of an 8-week overfeeding intervention (additional 40% of weight maintenance energy requirements) on healthy participants in an inpatient setting. This produced a significant increase in weight (5.52 ± 2.05 kg, $p < 0.0001$) and leptin levels (Figures S1K and S1L), but no rise in circulating GDF15. Indeed, at the end of the intervention, GDF15 levels were actually lower than at entry to the study (Figure S1M).

Consistent with these data, GDF15 levels were unchanged in mice fed an HFD for up to 7 days (Mouse Study 2), despite the mice manifesting the anticipated increases in fat and liver weight, and rises in plasma insulin and leptin (Figure S2).

Taken together, these data suggest that, unlike established hormonal regulators of energy homeostasis, "modest" overfeeding in humans and mice does not trigger GDF15 production.

GDF15 Levels Are Increased by Sustained Hypercaloric Loads

As GDF15 is a stress-responsive hormone, we wondered if more prolonged or severe nutritional stressors might be needed to induce its expression and secretion. To test this hypothesis, we undertook a prospective longitudinal study in mice fed either a CD or HFD from 9 weeks of age (Mouse Study 3). This resulted in progressive weight gain and fat mass in association with rising insulin and leptin levels (Figures 2A–2D). Glucose levels were initially similar in the two groups but rose significantly in the HFD-fed mice from week 4 onward (Figure 2E). GDF15 levels started to rise at the 4 week time point and were significantly higher in the HFD-fed mice from week 8 onward (Figure 2F).

In order to clarify the source of GDF15 in this context, we analyzed GDF15 mRNA expression in a range of tissues. GDF15 expression increased in liver, white epididymal adipose tissue (WAT), and even more strikingly in brown adipose tissue (BAT), but not in subcutaneous inguinal fat, kidney, and skeletal muscle (expression was very low in the latter) (Figure 2G).

GDF15 Expression Is Regulated by the Cellular Integrated Stress Response

Next we sought to establish why GDF15 expression was induced in these tissues in this context. Prior work had suggested that ATF4 and CHOP, key transcriptional regulators of the ISR, might be involved (Suzuki et al., 2017). The ISR is a cell-autonomous integrator of diverse cellular stresses, so we began by documenting changes in GDF15 mRNA in mouse embryonic fibroblasts (MEFs) treated with a range of well-characterized stressors (Figure 3). These included cobalt chloride, a chemical mimic of hypoxia, which acts by stabilizing hypoxia inducible factor 1a (HIF-1a); unfolded protein response (UPR) inducers thapsigargin, an inhibitor of sarcoplasmic/endoplasmic reticulum Ca^{2+}-ATPase (SERCA) and tunicamycin, an inhibitor of N-linked glycosylation, both of which perturb protein folding; and histidinol, an inhibitor of histidyl tRNA synthetase, which mimics amino acid deprivation. All these agents caused a significant and robust induction of GDF15 mRNA expression, albeit to varying extents, with thapsigargin being the most potent (Figure 3A). To confirm that GDF15 can be similarly upregulated in other cell types, we documented stress-induced changes in GDF15 expression in a range of human cell lines, as well as in 3T3-L1 preadipocytes (Figures 3B and S3A–S3C). In each case, GDF15 expression was increased with the level of induction ranging from 2- to 30-fold.

All of the stress-inducing agents used here can trigger phosphorylation of EIF2α by one of four known EIF2α kinases (Harding et al., 2000a, 2000b; Koumenis et al., 2002; Taniuchi et al., 2016), so we went on to confirm that this did occur at the concentrations and over the time frames used in our study (Figure 3C). Indeed, in addition to the stress-induced phosphorylation of EIF2α at Ser51, the protein expression of both the downstream targets, ATF4 and CHOP, was increased, albeit to a different extent, with tunicamycin and thapsigargin being the most potent inducers.

Next, we used a combination of inhibitors and KO or knockdown MEF lines to test the roles of various elements of the ISR pathway in the regulation of GDF15. First, using the PERK inhibitor GSK2606414 (abbreviated as GSK in Figure 1) or the EIF2α inhibitor ISRIB (abbreviated as ISR in Figure 1), we demonstrated that the tunicamycin-mediated induction of GDF15 was significantly reduced (Figure 3D), and that this correlated with a reduction in the activation of the ISR pathway, as judged by decreased ATF4 and CHOP expression (Figure S3D). We also found that the tunicamycin-induced expression of GDF15 was abolished in MEFs harboring a mutation at the key phosphorylation site on EIF2α (Ser51-Ala) required for ISR activation (Figure 3E).

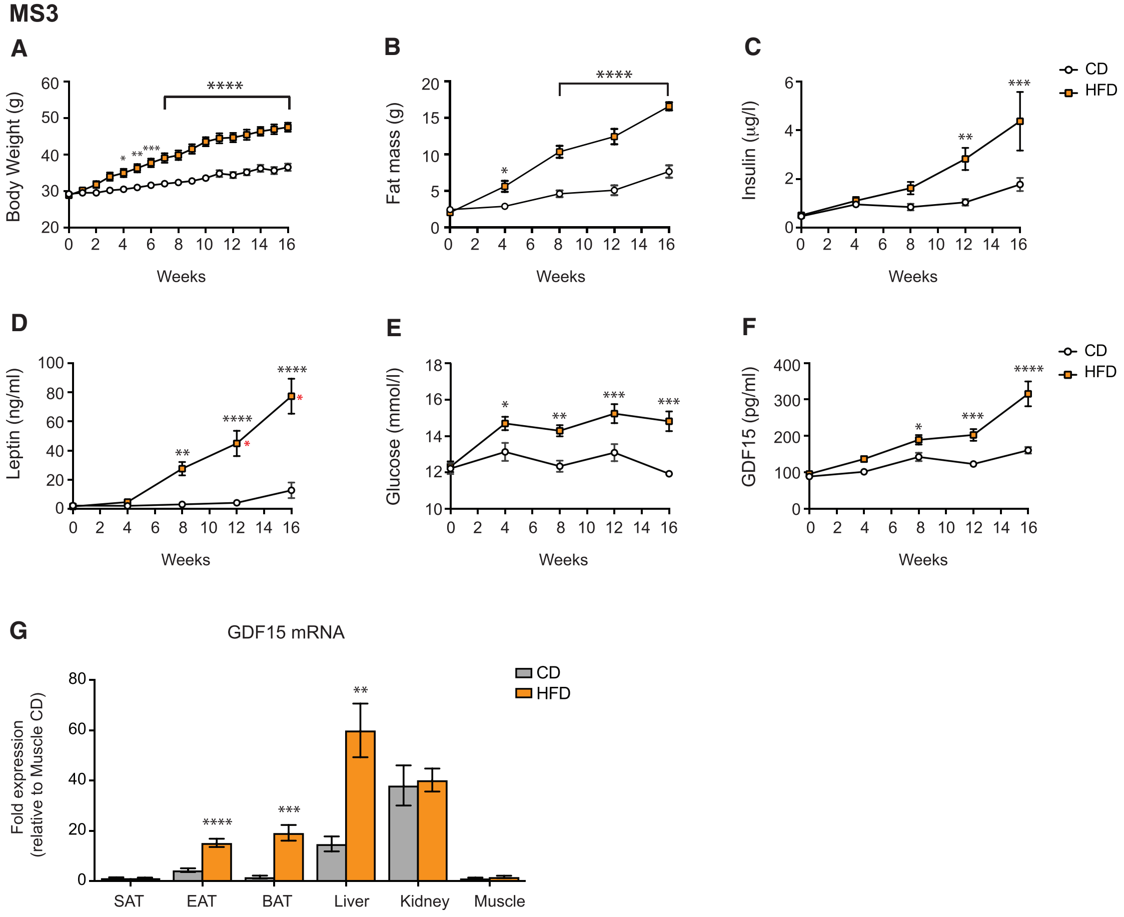

Figure 2. GDF15 Is Upregulated by Long-Term High-Fat Feeding in Mice

(A–F) Mouse Study 3 (MS3): C57BL/6J male mice (aged 9 weeks) were fed a chow (CD) or high-fat diet (HFD) for 16 weeks. (A) Body weight was recorded weekly (CD, n = 7; HFD, n = 8), while (B) fat mass and (C) insulin, (D) leptin, (E) glucose, and (F) GDF15 concentrations were determined at 0, 4, 8, 12 (CD, n = 9–11; HFD, n = 10–12), and 16 weeks (CD, n = 7; HFD, n = 8) (all after a 4 h fast). Data are expressed as mean ± SEM. *$p < 0.05$, **$p < 0.01$, ***$p < 0.001$, ****$p < 0.0001$ by two-way ANOVA with Bonferroni multiple comparison post-test. The red asterisks in (D) denote time points at which some (1 out of 12 at 12 weeks and 3 out of 8 at 16 weeks) leptin values were above the assay detection limit (>100 ng/mL) and thus were set at 100 ng/mL.

(G) GDF15 mRNA expression in subcutaneous (SAT), epididymal (EAT), and brown (BAT) adipose tissue, liver, soleus muscle, and kidney isolated from C57BL/6J male mice fed a CD or HFD for 18 weeks (n = 6–8 mice/group). mRNA is presented as fold expression (mean ± SEM) relative to the chow-fed state from muscle (set at 1) and normalized to the geometric mean of B2M/36b4 gene expression. **$p < 0.01$, ***$p < 0.001$, ****$p < 0.0001$ by two-tailed Student's t test. See also Figures S2 and S4.

Similarly, moving downstream in the pathway, tunicamycin-induced GDF15 expression was abolished in ATF4 KO MEFs (Figures 3F and S3E). Furthermore, small interfering RNA (siRNA)-mediated knockdown of CHOP significantly reduced both basal and tunicamycin-induced GDF15 expression (Figures 3G and S3F). Coupled with the knowledge that circulating GDF15 acts via GFRAL in the hindbrain, this establishes GDF15 as a bona fide systemic endocrine signal of the ISR.

In order to establish if the ISR is indeed responsible for the observed induction of GDF15 in long-term HF-fed mice, we next evaluated expression of ATF4 and CHOP mRNA in the same panel of tissues assessed for GDF15 expression (Mouse Study 3). These data confirmed that ATF4 and CHOP mRNA were increased in the liver and BAT (ATF4 only), but interestingly not in WAT (Figures S3G and S3H), suggesting that the induction of GDF15 in WAT may involve other signaling pathways. HF feeding in mice leads to adipocyte cell death, particularly in epididymal fat (Strissel et al., 2007), and this has been shown to activate macrophages (Cinti et al., 2005). Furthermore, GDF15 was originally identified in activated macrophages (Bootcov et al., 1997), so we proceeded to check mRNA expression of a macrophage marker, F4/80, in the WAT and BAT samples. F4/80 mRNA increased in parallel with the changes in GDF15 mRNA (Figure S3I), so we sought to establish if GDF15 mRNA was being induced in macrophages, or other stromovascular fraction (SVF) cells, or in adipocytes themselves. The data suggest that GDF15 mRNA is induced in both fractions (Figure S3J). However, lipid-laden macrophages may "contaminate" the adipocyte fraction as the separation is based on flotation, so we went on to check for this by analyzing mRNA expression of Plin1 (an adipocyte marker) and F4/80 in each of the fractions. These data suggest that macrophages are present in the adipocyte fraction, so it remains possible that the apparent increase in GDF15 mRNA

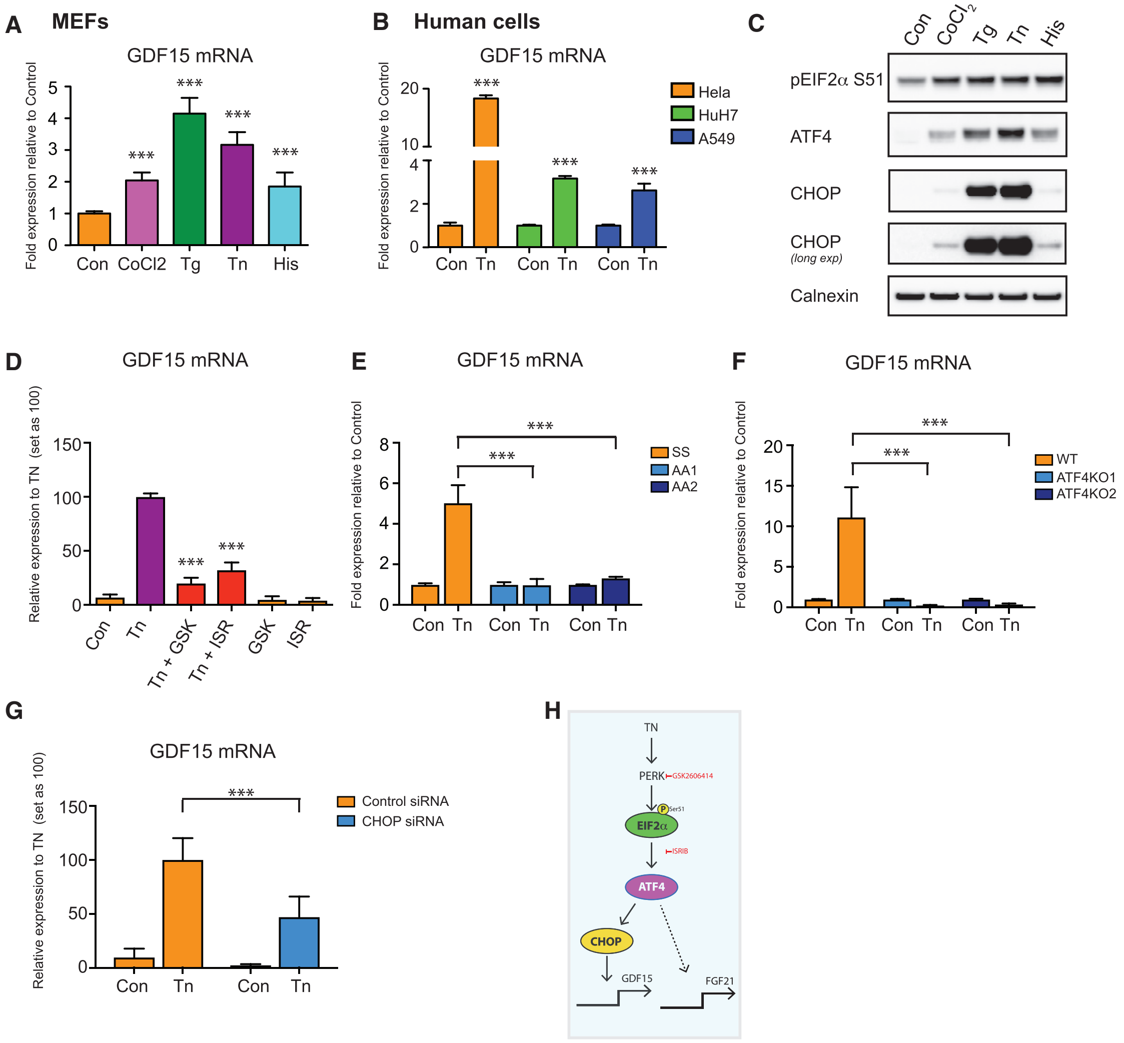

Figure 3. GDF15 Expression Is Regulated by the Cellular ISR Pathway

(A and C) GDF15 mRNA expression (A) and immunoblot analysis (C) of ISR components in wild-type (WT) mouse embryonic fibroblasts (MEFs) treated with vehicle control (Con), cobalt chloride (CoCl2, 625 μM), thapsigargin (Tg, 1 μM), tunicamycin (Tn 5 μg/mL), or L-Histidinol (His, 1 mM) for 6 h.

(B) GDF15 mRNA expression in human cell lines (HeLA, HuH7, and A549) treated with Tn (5 μg/mL) for 6 h.

(D) GDF15 mRNA expression in WT MEFs pre-treated for 1 h either with the PERK inhibitor GSK2606414 (GSK, 200 nM) or eIF2α inhibitor ISRIB (ISR, 100 nM), then co-treated with Tn (5 μg/mL) for a further 6 h.

(E–G) GDF15 mRNA expression (E) in EIF2α Ser51 (SS) or phospho mutant (AA) MEFs or (F) in ATF4 wild-type (WT) or ATF4 knockout (KO) MEFs and (G) in control siRNA and CHOP siRNA transfected WT MEFs treated with Tn (5 μg/mL) for 6 h.

(H) Diagram outlining pathway by which GDF15 and FGF21 expression is regulated by TN. mRNA expression is presented as fold expression relative to its respective control treatment for each cell type (set at 1) or TN-treated samples (set as 100) with normalization to HPRT gene expression in MEFs and GAPDH in human cells.

Data are expressed as mean ± SD from at least three independent experiments. ***$p < 0.001$ versus control (con) for (A) and (B), and versus TN stimulated for (D)–(G) by two-tailed Student's t test. Blots shown are representative of three independent experiments with Calnexin used as a loading control. See also Figures S3 and S4.

is largely coming from macrophages, though we cannot formally exclude a contribution from adipocytes themselves.

From these data, it is clear that in mice, GDF15 expression is responsive to chronic conditions of overnutrition that manifest with changes in GDF15 within adipose tissue (white and brown) and the liver. These findings are consistent with reported increases in GDF15 levels in ob/ob mice and in obese humans (Dostálová et al., 2009; Xiong et al., 2017), though the latter will require further careful analysis as Tsai et al. (2015) reported that in non-obese monozygotic twin pairs (n = 72 pairs), the twin with the higher GDF15 concentration had a lower BMI.

GDF15 Levels in Response to an Amino Acid Imbalanced Diet

Having demonstrated that nutritional overload can induce GDF15 expression, we wondered if other nutritional stresses

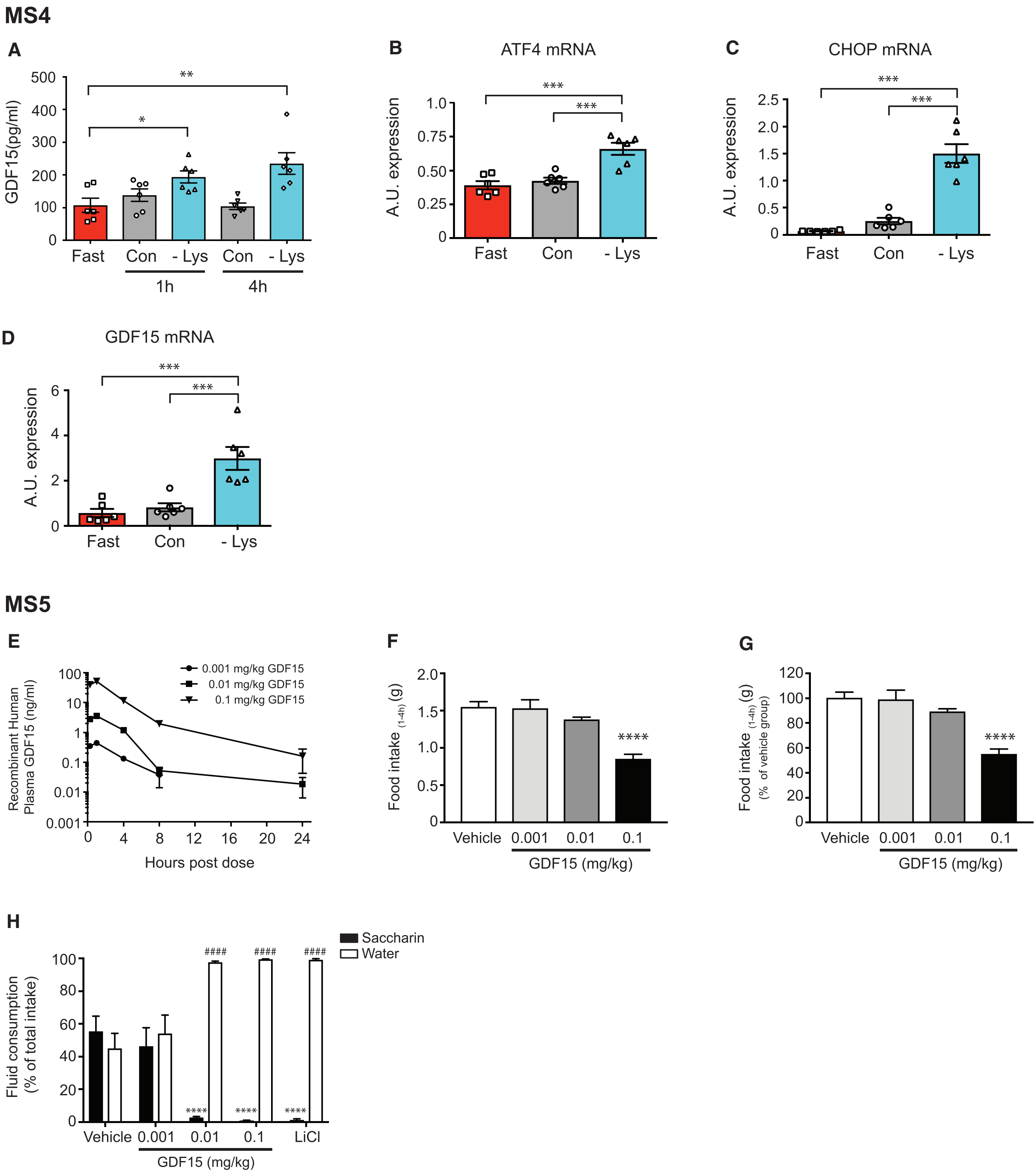

Figure 4. GDF15 Is Upregulated in Response to a Lysine-Deficient Diet and Induces Conditioned Taste Aversion in Mice

(A–D) Mouse Study 4 (MS4): (A) GDF15 serum concentrations and (B) ATF4, (C) CHOP, and (D) GDF15 mRNA expression in livers of 11- to 12-week-old female mice that were fasted overnight and then fed a control chow (Con) or lysine-deficient diet (–Lys) for 4 h. A blood sample was withdrawn at 1 h following the beginning of the meal. Serum and mRNA (4 h time point only) data are expressed as mean ± SEM (n = 6 mice per group) with mRNA normalized to B2M gene expression. *p < 0.05, **p < 0.01, ***p < 0.001 by one-way ANOVA.

(E) Mouse Study 5 (MS5): Circulating plasma GDF15 concentrations after a single dose of recombinant GDF15 in mice; dose response (n = 3/ group).

(legend continued on next page)

might have similar consequences. Previous studies have shown that diets deficient in essential amino acids can influence food intake and increase FGF21 levels in an ATF4-dependent manner (Gietzen et al., 2016; De Sousa-Coelho et al., 2012, 2013), so we wondered if such diets might have a comparable impact on GDF15 levels. This was of particular relevance as we had shown (Figure 3A) that pharmacological mimics of amino acid imbalance that activate the ISR increase GDF15 expression in cells. To test this hypothesis, mice were fasted overnight and then fed a lysine-deficient diet for 4 h (Mouse Study 4). This led to a marked increase in circulating GDF15 levels compared to chow-fed animals (Figure 4A). In keeping with activation of the ISR, ATF4, CHOP, and GDF15 mRNA were all significantly increased in the livers of these mice (Figures 4B–4D).

GDF15 Administration Results in Conditioned Taste Aversion

Reduced food intake has been shown to mediate most of the effects of GDF15 administration or overexpression on body weight (Emmerson et al., 2017; Macia et al., 2012; Mullican et al., 2017; Yang et al., 2017). Activation of the GDF15 receptor (GFRAL) has also been linked to subsequent cFos activation in the parabrachial nucleus (PBN), which has in turn been linked to appetite suppression in response to meal-related peptides, as well as ingestion of toxins, mimicked by lithium chloride and lipopolysaccharide (Carter et al., 2013; Hsu et al., 2017). Thus, we hypothesized that GDF15 administration might result in CTA, which occurs when an animal associates the taste of a normally favored food with symptoms caused by a concomitantly administered toxic or aversive substance.

In Mouse Study 5, we first assessed the ability of GDF15 to lower food intake in a concentration-dependent manner. A single subcutaneous injection of GDF15 in mice acutely increased plasma GDF15 concentration, with maximum exposures occurring 1 and 4 h post-treatment (Figure 4E). GDF15 treatment resulted in a corresponding dose-dependent reduction of food intake that reached statistical significance at the highest dose (0.1 mg/kg) (Figures 4F and 4G). We then addressed whether GDF15 induced CTA behavior using the two bottle saccharin preference test. Similar to the positive CTA control, lithium chloride, GDF15 treatment at 0.01 mg/kg and 0.1 mg/kg also reduced saccharin consumption and increased water consumption compared to vehicle control (Figure 4H).

These data demonstrate that acute administration of GDF15 can elicit an aversive response in rodents.

Nutritional Regulation of FGF21 Expression

Although its physiological function in humans is not clearly established, FGF21 is another putative systemic signal induced by the ISR (Fisher and Maratos-Flier, 2016; Salminen et al., 2017). In humans, plasma FGF21 levels did not change significantly following a mixed meal or oral glucose challenge, a week of total calorie restriction, or high-fat overfeeding in healthy volunteers (Figures S4A–S4D). In mice, short-term overfeeding induced a small increase in FGF21 levels, whereas prolonged HFD exposure was associated with a robust increase in FGF21 levels (Figures S4E and S4F). Interestingly, although FGF21 mRNA increased in similar tissues as GDF15, within WAT, FGF21 seemed to originate from adipocytes themselves rather than macrophages (Figures S4G and S4H). Lysine-deficient diet exposure in mice also elicited a significant increase in hepatic FGF21 mRNA (Figure S4I). In cells, FGF21 responses to activation of the ISR largely mirrored the GDF15 responses with one notable exception: exaggerated induction rather than amelioration of the FGF21 response to TN in CHOP knockdown cells (Figures S4J–S4N). These results suggest that while the ISR pathway regulates both hormones, the molecular mechanisms downstream of ATF4 are distinct. These data were corroborated by the finding that circulating FGF21 and hepatic FGF21 mRNA levels were significantly increased in mice following a 24 h fast, despite the lack of a significant change in ATF4 or CHOP mRNA expression (Figures S4O–S4R). Fasting-induced FGF21 expression is known to involve PPARα (Badman et al., 2007; Inagaki et al., 2007).

DISCUSSION

Elevating GDF15 levels by transgenic overexpression (Chrysovergis et al., 2014; Macia et al., 2012) or pharmacological administration in mice and nonhuman primates leads to a marked fall in body weight (Mullican et al., 2017). The principal aim of our work was to understand if and how GDF15 might be involved in physiological settings of under- and over-nutrition. To this end, we combined cellular studies with *in vivo* work in mice and humans to establish that GDF15 expression is highly responsive to activation of the ISR in a range of cell types and that its induction in this setting is dependent upon ATF4 and CHOP. The idea that cellular stress might be translated into a systemic response initially emerged from work in *C. elegans* where an induction of the mitochondrial unfolded protein response (UPR^{mt}) in neurons led to changes in mitochondria within physically distinct, non-innervated tissues (Durieux et al., 2011), but has more recently been supported by evidence linking FGF21 to the ISR (Salminen et al., 2017). Chung et al. (2017) also recently proposed that GDF15 could act as a ‘‘mitohormetic’’ signal of mitochondrial dysfunction. Our analysis is largely consistent with these data and provides compelling evidence of the induction of GDF15 in response to activation of the ISR.

As GDF15 administration causes weight loss and mice lacking GDF15 are prone to gain weight on an HFD, we determined whether GDF15 shares any features in common with known hormonal regulators of post-prandial satiety (e.g., enteroendocrine hormones such as GLP-1) or longer term hormonal regulators of nutrient stores (e.g., leptin). In contrast to GLP-1, and consistent with previous reports (Schernthaner-Reiter et al., 2016; Tsai et al., 2015), GDF15 did not respond acutely to a

(F and G) Cumulative food intake measured between 1 and 4 h post-GDF15 dose expressed as total grams (F) or percent (%) of vehicle control (G) (n = 7–8/group). Data are presented as mean ± SEM. ****p < 0.0001 versus vehicle by one-way ANOVA with Bonferroni multiple comparison post-test.

(H) Saccharin and water consumption during conditioned taste aversion test during GDF15 treatment (n = 8–16/group). Data are presented as mean ± SEM and analyzed using a two-way ANOVA with Bonferroni multiple comparison post-test to compare proportion of saccharin water and water consumption between groups of GDF15 or LiCl treatment to vehicle. ****(saccharin) or ####(water) p < 0.0001. See also Figure S4.

meal or a glucose load in humans. In mice fasted for 24 h, there was no change in circulating GDF15, whereas the predicted fall in leptin levels and rise in FGF21 levels was seen. In humans, 48 h of severe caloric restriction in lean healthy volunteers resulted in a significant but small increase in GDF15 concentrations. In healthy volunteers undergoing a 7 day total fast, GDF15 levels peaked at around ~180% of baseline by day 3 and then plateaued at around 118% at day 7. This early rise in GDF15 is in the opposite direction expected of a physiological regulator of energy balance and is more consistent with GDF15 being a marker of cell/tissue stress. The mechanisms whereby GDF15 levels start to return toward baseline with more prolonged fasting are unknown, but presumably reflect some sort of adaptation to the starved state.

In two separate studies, overfeeding of healthy humans with an ~48% excess of ingested calories for 1 week, or 40% for 8 weeks, did not increase GDF15 concentrations. Of note, in the longer study, conducted in an inpatient setting, GDF15 levels showed a small but significant fall (Figure S1M). Among possible explanations for this fall is the fact that in this inpatient study, smoking was not permitted. GDF15 levels are known to be positively associated with smoking status and it is possible that some participants quit smoking just prior to the study (Ho et al., 2012; Wu et al., 2012).

In contrast to the studies summarized above, we found that circulating GDF15 levels rose in long-term HF feeding studies in mice. Whether or not this is also true in humans will require further studies. As recently summarized by Tsai et al., the relationship between circulating GDF15 and obesity in humans is complex. GDF15 levels rise with age and are also induced by conditions commonly associated with obesity such as diabetes and cardiovascular disease (Tsai et al., 2018; Wollert et al., 2017). So while positive correlations between GDF15 and measures of adiposity have been reported in several small studies (Dostálová et al., 2009; Ho et al., 2012; Karczewska-Kupczewska et al., 2012; Kempf et al., 2012; Vila et al., 2011), GDF15 was shown to be inversely correlated with BMI in non-obese monozygotic twin pairs (Tsai et al., 2015). It is plausible that an inherent genetically determined increase in GDF15 levels or one induced by another cell stressor/disease might result in weight loss, and thus confound straightforward correlations between BMI and GDF15 levels.

Ravussin et al. have drawn attention to the likely existence of leptin-independent signals of the obese state that might serve to restrain the indefinite progression of a state of positive energy balance and ever increasing obesity (Ravussin et al., 2014). The fact that mice lacking GDF15 become more obese on an HFD than wild-type mice suggests that GDF15 might at least contribute to that signal (Hsu et al., 2017; Tran et al., 2018). Our studies show that while short-term overfeeding does not increase GDF15, more sustained states of caloric excess will raise circulating GDF15 levels.

What is the source of the elevated levels of the GDF15 seen in the overnourished state? In mice, GDF15 mRNA is increased in liver and BAT, and in certain WAT depots, such as epididymal, by 18 weeks of HFD, but not in kidney or muscle. In adipose tissue it appears that most of the GDF15 mRNA is likely to be coming from infiltrating macrophages, whereas our initial analyses suggest that FGF21 mRNA is more robustly induced in adipocytes themselves. Macrophages are frequently cited as mediating many of the adverse metabolic consequences of overnutrition such as insulin resistance, through their production of a range of cytokine-like molecules. In the case of GDF15, it appears that these cells produce a circulating product that in this particular context might be beneficial to the organism.

We also show GDF15 is significantly induced by another nutritional stress, namely a lysine-deficient diet. Interestingly, involvement of the ISR in response to perturbations affecting amino acid provision is conserved as far back as yeast (Hinnebusch, 2005), where the response is clearly cell autonomous. In metazoans, this response expanded to encompass rectifying responses to other cell-autonomous perturbations such as the UPR, hypoxia, viral infections, and iron deficiency (Pakos-Zebrucka et al., 2016). The link to the GDF15-GFRAL axis suggests that the ISR may now also have gained an endocrine component potentially involving an aversive response instructing the animal to change its foraging pattern. In some settings this is likely to be advantageous to the animal, though there are clearly exceptions where it is not, such as in cancer cachexia, where GDF15 levels can be as high as 40,000 pg/mL (Johnen et al., 2007; Welsh et al., 2003).

What, then, are the consequences of induction of this hormone? GDF15 has been shown to reduce food intake in various species and to alter food choice. While it has been speculated that GDF15 may produce an aversive response (O'Rahilly, 2017), this had not previously been formally demonstrated. Using a CTA paradigm, we show that mice exposed to GDF15 in association with a saccharin taste will avoid saccharin-containing drinking water in future exposures. CTA is classically elicited by injection of lithium chloride and is also typical of agents that are known to produce nausea in humans. In this context, GDF15 contrasts with leptin, which reduces food intake but does not produce CTA (Thiele et al., 1997). The lateral PBN, medial thalamus, and basolateral nucleus of the amygdala are essential for both acquisition and retention of CTA, with CGRP-expressing neurons of the PBN playing an essential role (Palmiter, 2018). Ascending pathways from the AP and NTS make substantial connections to the lateral PBN. It is therefore likely, though yet to be formally established, that GFRAL-expressing neurons of the caudal hindbrain project to the PBN.

In summary, GDF15 appears to be an endocrine signal that can be produced by almost any cell type in response to activation of the ISR and presumably other signals. Our data suggest that "nutritional stress," whether induced by sustained overnutrition as exemplified by prolonged HF feeding in mice or by provision of an amino acid imbalanced diet, leads to increased circulating GDF15 levels. We suggest that this might send an aversive endocrine signal to the brain, though we acknowledge that further work is needed to verify this hypothesis. If GDF15 is playing a role in restraining progressive weight gain, this suggests that it might have a role in the therapy of obesity. While its production of CTA might seem to suggest that it would be poorly tolerated in humans, it is premature to conclude that this should prevent its exploration as a possible obesity therapeutic. GLP-1-based drugs are licensed for the treatment of obesity, yet they too produce a CTA response in rodents and activate the lateral PBN (Thiele et al., 1997). With careful titration, most humans can tolerate therapeutic doses of GLP-1 receptor agonists,

though nausea and vomiting do lead to its cessation in a significant number. Thus, these data support the burgeoning interest in GFRAL, the GDF15 receptor, as an attractive therapeutic target in so far as it has a highly tissue-specific expression with actions of GDF15 at sites other than the caudal brain stem seemingly unlikely. Studies of the effects of GDF15 in humans are eagerly anticipated.

Limitations of Study

Limitations of our studies include the fact that while we have shown that HF and lysine-deficient diets can induce GDF15, and that when administered at pharmacological doses, GDF15 can induce an aversive response, we have yet to formally demonstrate that this also occurs when GDF15 is induced endogenously. One might also argue that the small increases in GDF15 we detected in response to extreme fasting in humans are counter-intuitive; however, we speculate that in these circumstances, in addition to causing food aversion, GDF15 might also cause malaise, which might encourage an animal to rest and conserve energy.

STAR★METHODS

Detailed methods are provided in the online version of this paper and include the following:

- KEY RESOURCES TABLE
- CONTACT FOR REAGENT AND RESOURCE SHARING
- EXPERIMENTAL MODEL AND SUBJECT DETAILS
  - Human Subjects
  - Mouse Studies
  - Eukaryotic Cell Lines
- METHOD DETAILS
  - Human Studies
  - Mouse Studies
  - Eukaryotic Cell Lines
  - siRNA Transfection and Knockdown CHOP
  - RNA Isolation/cDNA Synthesis/Q-PCR
  - Serum and Media Analysis
  - Immunoblotting
- QUANTIFICATION AND STATISTICAL ANALYSIS

SUPPLEMENTAL INFORMATION

Supplemental Information includes four figures and can be found with this article online at https://doi.org/10.1016/j.cmet.2018.12.016.

ACKNOWLEDGMENTS

The authors would like to thank all the volunteers for participating in the various studies. We would also like to thank Steffen Brufladt, Emelie Nilsen, Egil I. Johansen, Marte Valde, and Kristoffer J Kolnes for conducting the 7 day fasting study (HS4); Laurent Parry, Mehdi Djelloul-Mazouz, and Yoann Delorme for technical assistance with MS4; and Joshua Smith and the nursing, dietary, and laboratory personnel of the Translational Research Institute for Metabolism and Diabetes, Florida, for help with HS6. We thank Peter Barker, Keith Burling, and other members of the NIHR Cambridge Biochemical Assay Laboratory (CBAL) for GDF15 and metabolite analyses. HS6 is supported in part by a grant from the US Department of Agriculture: 2010-34323-21052. Mouse Studies 1, 2, and 3 are supported by The Disease Model Core, part of the MRC Metabolic Diseases Unit (MRC_MC_UU_12012/5), and Wellcome Trust Strategic Award (100574/Z/12/Z). D.B.S. and S.O.R. are supported by the Wellcome Trust (WT 107064 and WT 095515/Z/11/Z), the MRC Metabolic Disease Unit (MRC_MC_UU_12012.1), and The National Institute for Health Research (NIHR) Cambridge Biomedical Research Centre and NIHR Rare Disease Translational Research Collaboration. A.P.C., D. Rimmington, and I.C. are supported by the Medical Research Council (MRC Metabolic Diseases Unit [MRC_MC_UU_12012.1]). D. Ron is supported by a Wellcome Trust Principal Research Fellowship (Wellcome 200848/Z/16/Z) and a Wellcome Trust Strategic Award to the Cambridge Institute for Medical Research (Wellcome 100140). A.V.-P. and S.V. are supported by the BHF (RG/12/13/29853) and MRC (MC_UU_12012/2). I.S.F. was supported by the Wellcome Trust (098497/Z/12/Z), European Research Council, NIHR Cambridge Biomedical Research Centre, and Bernard Wolfe Health Neuroscience Endowment. A.M. is supported by a studentship from the Experimental Medicine Training Initiative/AstraZeneca. G.P.R. was supported by an Addenbrooke's Charitable Trust/Evelyn Trust Cambridge Clinical Research Fellowship (16-69), an EFSD project grant, and a Royal College of Surgeons Research Fellowship. C.L.M. is supported by the Diabetes UK Harry Keen intermediate clinical fellowship (17/0005712). F.M.G. and F.R. are supported by the MRC (MRC_MC_UU_12012/3), Wellcome Trust (106262/Z/14/Z and 106263/Z/14/Z), and research grants from Medimmune.

AUTHOR CONTRIBUTIONS

Overall conceptualization by A.P.C., D.B.S., S.O.R., S.P., A.A.-G., and A.M. A.-C.M., H.P.H., D. Ron, C.J.H., I.S.F., L.M.R., G.A.B., A.V.-P., F.R., F.M.G., P.F., S.R.S., P.B.J., A.J.K., J.J., D.B., Z.W., and B.B.Z. also contributed to conceptualization of individual experiments and studies included in this body of work. Experimental investigation by S.P., A.A.-G., A.M., A.D., D. Rimmington, A.-C.M., E.L.M., I.C., S.V., G.P.R., C.L.M., A.P.L., L.M.S., S.A. Parsons, S.R.S., R.M.W., and S.A. Parry. S.P., A.A.-G., A.M., S.V., L.M.S., S.A. Parsons, and A.P.C. analyzed data. S.P., A.P.C., D.B.S., and S.O.R. wrote the paper, which was reviewed and edited by all the authors.

DECLARATION OF INTERESTS

S.O.R. is an employee of the University of Cambridge and has provided remunerated consultancy services to the following pharmaceutical companies with an active or potential interest in GDF15: Pfizer, AstraZeneca, MedImmune, and Novo-Nordisk. A.P.L., D.B., Z.W., and B.B.Z. were employed by Pfizer during the reported studies.

Received: July 20, 2018
Revised: October 10, 2018
Accepted: December 17, 2018
Published: January 10, 2019

REFERENCES

Appierto, V., Tiberio, P., Villani, M.G., Cavadini, E., and Formelli, F. (2009). PLAB induction in fenretinide-induced apoptosis of ovarian cancer cells occurs via a ROS-dependent mechanism involving ER stress and JNK activation. Carcinogenesis *30*, 824–831.

Badman, M.K., Pissios, P., Kennedy, A.R., Koukos, G., Flier, J.S., and Maratos-Flier, E. (2007). Hepatic fibroblast growth factor 21 is regulated by PPARalpha and is a key mediator of hepatic lipid metabolism in ketotic states. Cell Metab. *5*, 426–437.

Bootcov, M.R., Bauskin, A.R., Valenzuela, S.M., Moore, A.G., Bansal, M., He, X.Y., Zhang, H.P., Donnellan, M., Mahler, S., Pryor, K., et al. (1997). MIC-1, a novel macrophage inhibitory cytokine, is a divergent member of the TGF-beta superfamily. Proc. Natl. Acad. Sci. USA *94*, 11514–11519.

Böttner, M., Suter-Crazzolara, C., Schober, A., and Unsicker, K. (1999). Expression of a novel member of the TGF-beta superfamily, growth/differentiation factor-15/macrophage-inhibiting cytokine-1 (GDF-15/MIC-1) in adult rat tissues. Cell Tissue Res. *297*, 103–110.

Bray, G.A., Smith, S.R., de Jonge, L., Xie, H., Rood, J., Martin, C.K., Most, M., Brock, C., Mancuso, S., and Redman, L.M. (2012). Effect of dietary protein content on weight gain, energy expenditure, and body composition during overeating: a randomized controlled trial. JAMA *307*, 47–55.

Bray, G.A., Redman, L.M., de Jonge, L., Covington, J., Rood, J., Brock, C., Mancuso, S., Martin, C.K., and Smith, S.R. (2015). Effect of protein overfeeding on energy expenditure measured in a metabolic chamber. Am. J. Clin. Nutr. *101*, 496–505.

Brown, D.A., Ward, R.L., Buckhaults, P., Liu, T., Romans, K.E., Hawkins, N.J., Bauskin, A.R., Kinzler, K.W., Vogelstein, B., and Breit, S.N. (2003). MIC-1 serum level and genotype: associations with progress and prognosis of colorectal carcinoma. Clin. Cancer Res. *9*, 2642–2650.

Carter, M.E., Soden, M.E., Zweifel, L.S., and Palmiter, R.D. (2013). Genetic identification of a neural circuit that suppresses appetite. Nature *503*, 111–114.

Chaveroux, C., Bruhat, A., Carraro, V., Jousse, C., Averous, J., Maurin, A.-C., Parry, L., Mesclon, F., Muranishi, Y., Cordelier, P., et al. (2016). Regulating the expression of therapeutic transgenes by controlled intake of dietary essential amino acids. Nat. Biotechnol. *34*, 746–751.

Chrysovergis, K., Wang, X., Kosak, J., Lee, S.H., Kim, J.S., Foley, J.F., Travlos, G., Singh, S., Baek, S.J., and Eling, T.E. (2014). NAG-1/GDF-15 prevents obesity by increasing thermogenesis, lipolysis and oxidative metabolism. Int. J. Obes. *38*, 1555–1564.

Chung, H.K., Ryu, D., Kim, K.S., Chang, J.Y., Kim, Y.K., Yi, H.S., Kang, S.G., Choi, M.J., Lee, S.E., Jung, S.B., et al. (2017). Growth differentiation factor 15 is a myomitokine governing systemic energy homeostasis. J. Cell Biol. *216*, 149–165.

Cinti, S., Mitchell, G., Barbatelli, G., Murano, I., Ceresi, E., Faloia, E., Wang, S., Fortier, M., Greenberg, A.S., and Obin, M.S. (2005). Adipocyte death defines macrophage localization and function in adipose tissue of obese mice and humans. J. Lipid Res. *46*, 2347–2355.

Corre, J., Hébraud, B., and Bourin, P. (2013). Concise review: growth differentiation factor 15 in pathology: a clinical role? Stem Cells Transl. Med. *2*, 946–952.

De Sousa-Coelho, A.L., Marrero, P.F., and Haro, D. (2012). Activating transcription factor 4-dependent induction of FGF21 during amino acid deprivation. Biochem. J. *443*, 165–171.

De Sousa-Coelho, A.L., Relat, J., Hondares, E., Pérez-Martí, A., Ribas, F., Villarroya, F., Marrero, P.F., and Haro, D. (2013). FGF21 mediates the lipid metabolism response to amino acid starvation. J. Lipid Res. *54*, 1786–1797.

Ding, Q., Mracek, T., Gonzalez-Muniesa, P., Kos, K., Wilding, J., Trayhurn, P., and Bing, C. (2009). Identification of macrophage inhibitory cytokine-1 in adipose tissue and its secretion as an adipokine by human adipocytes. Endocrinology *150*, 1688–1696.

Dostálová, I., Roubícek, T., Bártlová, M., Mráz, M., Lacinová, Z., Haluzíková, D., Kaválková, P., Matoulek, M., Kasalický, M., and Haluzík, M. (2009). Increased serum concentrations of macrophage inhibitory cytokine-1 in patients with obesity and type 2 diabetes mellitus: the influence of very low calorie diet. Eur. J. Endocrinol. *161*, 397–404.

Durieux, J., Wolff, S., and Dillin, A. (2011). The cell-non-autonomous nature of electron transport chain-mediated longevity. Cell *144*, 79–91.

Emmerson, P.J., Wang, F., Du, Y., Liu, Q., Pickard, R.T., Gonciarz, M.D., Coskun, T., Hamang, M.J., Sindelar, D.K., Ballman, K.K., et al. (2017). The metabolic effects of GDF15 are mediated by the orphan receptor GFRAL. Nat. Med. *23*, 1215–1219.

Fairlie, W.D., Moore, A.G., Bauskin, A.R., Russell, P.K., Zhang, H.P., and Breit, S.N. (1999). MIC-1 is a novel TGF-beta superfamily cytokine associated with macrophage activation. J. Leukoc. Biol. *65*, 2–5.

Fisher, F.M., and Maratos-Flier, E. (2016). Understanding the physiology of FGF21. Annu. Rev. Physiol. *78*, 223–241.

Fujita, Y., Taniguchi, Y., Shinkai, S., Tanaka, M., and Ito, M. (2016). Secreted growth differentiation factor 15 as a potential biomarker for mitochondrial dysfunctions in aging and age-related disorders. Geriatr. Gerontol. Int. *16* (*Suppl 1*), 17–29.

Gietzen, D.W., Anthony, T.G., Fafournoux, P., Maurin, A.C., Koehnle, T.J., and Hao, S. (2016). Measuring the ability of mice to sense dietary essential amino acid deficiency: the importance of amino acid status and timing. Cell Rep. *16*, 2049–2050.

Harding, H.P., Novoa, I., Zhang, Y., Zeng, H., Wek, R., Schapira, M., and Ron, D. (2000a). Regulated translation initiation controls stress-induced gene expression in mammalian cells. Mol. Cell *6*, 1099–1108.

Harding, H.P., Zhang, Y., Bertolotti, A., Zeng, H., and Ron, D. (2000b). Perk is essential for translational regulation and cell survival during the unfolded protein response. Mol. Cell *5*, 897–904.

Harding, H.P., Zhang, Y., Zeng, H., Novoa, I., Lu, P.D., Calfon, M., Sadri, N., Yun, C., Popko, B., Paules, R., et al. (2003). An integrated stress response regulates amino acid metabolism and resistance to oxidative stress. Mol. Cell *11*, 619–633.

Hinnebusch, A.G. (2005). Translational regulation of *GCN4* and the general amino acid control of yeast. Annu. Rev. Microbiol. *59*, 407–450.

Ho, J.E., Mahajan, A., Chen, M.H., Larson, M.G., McCabe, E.L., Ghorbani, A., Cheng, S., Johnson, A.D., Lindgren, C.M., Kempf, T., et al. (2012). Clinical and genetic correlates of growth differentiation factor 15 in the community. Clin. Chem. *58*, 1582–1591.

Hsiao, E.C., Koniaris, L.G., Zimmers-Koniaris, T., Sebald, S.M., Huynh, T.V., and Lee, S.J. (2000). Characterization of growth-differentiation factor 15, a transforming growth factor beta superfamily member induced following liver injury. Mol. Cell. Biol. *20*, 3742–3751.

Hsu, J.Y., Crawley, S., Chen, M., Ayupova, D.A., Lindhout, D.A., Higbee, J., Kutach, A., Joo, W., Gao, Z., Fu, D., et al. (2017). Non-homeostatic body weight regulation through a brainstem-restricted receptor for GDF15. Nature *550*, 255–259.

Inagaki, T., Dutchak, P., Zhao, G., Ding, X., Gautron, L., Parameswara, V., Li, Y., Goetz, R., Mohammadi, M., Esser, V., et al. (2007). Endocrine regulation of the fasting response by PPARalpha-mediated induction of fibroblast growth factor 21. Cell Metab. *5*, 415–425.

Johnen, H., Lin, S., Kuffner, T., Brown, D.A., Tsai, V.W.-W., Bauskin, A.R., Wu, L., Pankhurst, G., Jiang, L., Junankar, S., et al. (2007). Tumor-induced anorexia and weight loss are mediated by the TGF-β superfamily cytokine MIC-1. Nat. Med. *13*, 1333–1340.

Karczewska-Kupczewska, M., Kowalska, I., Nikolajuk, A., Adamska, A., Otziomek, E., Gorska, M., and Straczkowski, M. (2012). Hyperinsulinemia acutely increases serum macrophage inhibitory cytokine-1 concentration in anorexia nervosa and obesity. Clin. Endocrinol. (Oxf.) *76*, 46–50.

Kempf, T., Horn-Wichmann, R., Brabant, G., Peter, T., Allhoff, T., Klein, G., Drexler, H., Johnston, N., Wallentin, L., and Wollert, K.C. (2007). Circulating concentrations of growth-differentiation factor 15 in apparently healthy elderly individuals and patients with chronic heart failure as assessed by a new immunoradiometric sandwich assay. Clin. Chem. *53*, 284–291.

Kempf, T., Guba-Quint, A., Torgerson, J., Magnone, M.C., Haefliger, C., Bobadilla, M., and Wollert, K.C. (2012). Growth differentiation factor 15 predicts future insulin resistance and impaired glucose control in obese nondiabetic individuals: results from the XENDOS trial. Eur. J. Endocrinol. *167*, 671–678.

Kleinert, M., Clemmensen, C., Sjøberg, K.A., Carl, C.S., Jeppesen, J.F., Wojtaszewski, J.F.P., Kiens, B., and Richter, E.A. (2018). Exercise increases circulating GDF15 in humans. Mol. Metab. *9*, 187–191.

Koumenis, C., Naczki, C., Koritzinsky, M., Rastani, S., Diehl, A., Sonenberg, N., Koromilas, A., and Wouters, B.G. (2002). Regulation of protein synthesis by hypoxia via activation of the endoplasmic reticulum kinase PERK and phosphorylation of the translation initiation factor eIF2alpha. Mol. Cell. Biol. *22*, 7405–7416.

Lawton, L.N., Bonaldo, M.F., Jelenc, P.C., Qiu, L., Baumes, S.A., Marcelino, R.A., de Jesus, G.M., Wellington, S., Knowles, J.A., Warburton, D., et al. (1997). Identification of a novel member of the TGF-beta superfamily highly expressed in human placenta. Gene *203*, 17–26.

Macia, L., Tsai, V.W.-W.W., Nguyen, A.D., Johnen, H., Kuffner, T., Shi, Y.-C.C., Lin, S., Herzog, H., Brown, D.A., Breit, S.N., and Sainsbury, A. (2012). Macrophage inhibitory cytokine 1 (MIC-1/GDF15) decreases food intake, body weight and improves glucose tolerance in mice on normal & obesogenic diets. PLoS One *7*, e34868.

Marjono, A.B., Brown, D.A., Horton, K.E., Wallace, E.M., Breit, S.N., and Manuelpillai, U. (2003). Macrophage inhibitory cytokine-1 in gestational tissues

and maternal serum in normal and pre-eclamptic pregnancy. Placenta *24*, 100–106.

Maurin, A.C., Jousse, C., Averous, J., Parry, L., Bruhat, A., Cherasse, Y., Zeng, H., Zhang, Y., Harding, H.P., Ron, D., and Fafournoux, P. (2005). The GCN2 kinase biases feeding behavior to maintain amino acid homeostasis in omnivores. Cell Metab. *1*, 273–277.

Mifflin, M.D., St Jeor, S.T., Hill, L.A., Scott, B.J., Daugherty, S.A., and Koh, Y.O. (1990). A new predictive equation for resting energy expenditure in healthy individuals. Am. J. Clin. Nutr. *51*, 241–247.

Mullican, S.E., and Rangwala, S.M. (2018). Uniting GDF15 and GFRAL: therapeutic opportunities in obesity and beyond. Trends Endocrinol. Metab. *29*, 560–570.

Mullican, S.E., Lin-Schmidt, X., Chin, C.N., Chavez, J.A., Furman, J.L., Armstrong, A.A., Beck, S.C., South, V.J., Dinh, T.Q., Cash-Mason, T.D., et al. (2017). GFRAL is the receptor for GDF15 and the ligand promotes weight loss in mice and nonhuman primates. Nat. Med. *23*, 1150–1157.

O'Rahilly, S. (2017). GDF15-from biomarker to allostatic hormone. Cell Metab. *26*, 807–808.

Oslowski, C.M., and Urano, F. (2011). Measuring ER stress and the unfolded protein response using mammalian tissue culture system. Methods Enzymol. *490*, 71–92.

Pakos-Zebrucka, K., Koryga, I., Mnich, K., Ljujic, M., Samali, A., and Gorman, A.M. (2016). The integrated stress response. EMBO Rep. *17*, 1374–1395.

Palmiter, R.D. (2018). The parabrachial nucleus: CGRP neurons function as a general alarm. Trends Neurosci. *41*, 280–293.

Park, S.H., Choi, H.J., Yang, H., Do, K.H., Kim, J., Kim, H.H., Lee, H., Oh, C.G., Lee, D.W., and Moon, Y. (2012). Two in-and-out modulation strategies for endoplasmic reticulum stress-linked gene expression of pro-apoptotic macrophage-inhibitory cytokine 1. J. Biol. Chem. *287*, 19841–19855.

Ravussin, Y., Leibel, R.L., and Ferrante, A.W., Jr. (2014). A missing link in body weight homeostasis: the catabolic signal of the overfed state. Cell Metab. *20*, 565–572.

Roberts, G.P., Kay, R.G., Howard, J., Hardwick, R.H., Reimann, F., and Gribble, F.M. (2018). Gastrectomy with Roux-en-Y reconstruction as a lean model of bariatric surgery. Surg. Obes. Relat. Dis. *14*, 562–568.

Salminen, A., Kaarniranta, K., and Kauppinen, A. (2017). Integrated stress response stimulates FGF21 expression: Systemic enhancer of longevity. Cell. Signal. *40*, 10–21.

Schernthaner-Reiter, M.H., Kasses, D., Tugendsam, C., Riedl, M., Peric, S., Prager, G., Krebs, M., Promintzer-Schifferl, M., Clodi, M., Luger, A., and Vila, G. (2016). Growth differentiation factor 15 increases following oral glucose ingestion: effect of meal composition and obesity. Eur. J. Endocrinol. *175*, 623–631.

Scheuner, D., Song, B., McEwen, E., Liu, C., Laybutt, R., Gillespie, P., Saunders, T., Bonner-Weir, S., and Kaufman, R.J. (2001). Translational control is required for the unfolded protein response and in vivo glucose homeostasis. Mol. Cell *7*, 1165–1176.

Schofield, W.N. (1985). Predicting basal metabolic rate, new standards and review of previous work. Hum. Nutr. Clin. Nutr. *39* (*Suppl 1*), 5–41.

Strissel, K.J., Stancheva, Z., Miyoshi, H., Perfield, J.W., 2nd, DeFuria, J., Jick, Z., Greenberg, A.S., and Obin, M.S. (2007). Adipocyte death, adipose tissue remodeling, and obesity complications. Diabetes *56*, 2910–2918.

Suzuki, T., Gao, J., Ishigaki, Y., Kondo, K., Sawada, S., Izumi, T., Uno, K., Kaneko, K., Tsukita, S., Takahashi, K., et al. (2017). ER stress protein CHOP mediates insulin resistance by modulating adipose tissue macrophage polarity. Cell Rep. *18*, 2045–2057.

Taniuchi, S., Miyake, M., Tsugawa, K., Oyadomari, M., and Oyadomari, S. (2016). Integrated stress response of vertebrates is regulated by four eIF2α kinases. Sci. Rep. *6*, 32886.

Thiele, T.E., Van Dijk, G., Campfield, L.A., Smith, F.J., Burn, P., Woods, S.C., Bernstein, I.L., and Seeley, R.J. (1997). Central infusion of GLP-1, but not leptin, produces conditioned taste aversions in rats. Am. J. Physiol. *272*, R726–R730.

Tran, T., Yang, J., Gardner, J., and Xiong, Y. (2018). GDF15 deficiency promotes high fat diet-induced obesity in mice. PLoS One *13*, e0201584.

Tsai, V.W.-W., Macia, L., Johnen, H., Kuffner, T., Manadhar, R., Jørgensen, S.B., Lee-Ng, K.K.M., Zhang, H.P., Wu, L., Marquis, C.P., et al. (2013). TGF-b superfamily cytokine MIC-1/GDF15 is a physiological appetite and body weight regulator. PLoS One *8*, e55174.

Tsai, V.W.-W., Manandhar, R., Jorgensen, S.B., Lee-Ng, K.K.M., Zhang, H.P., Marquis, C.P., Jiang, L., Husaini, Y., Lin, S., Sainsbury, A., et al. (2014). The anorectic actions of the TGFβ cytokine MIC-1/GDF15 require an intact brainstem area postrema and nucleus of the solitary tract. PLoS One *9*, 1–10.

Tsai, V.W.-W., Macia, L., Feinle-Bisset, C., Manandhar, R., Astrup, A., Raben, A., Lorenzen, J.K., Schmidt, P.T., Wiklund, F., Pedersen, N.L., et al. (2015). Serum levels of human MIC-1/GDF15 vary in a diurnal pattern, do not display a profile suggestive of a satiety factor and are related to BMI. PLoS One *10*, e0133362.

Tsai, V.W.W., Lin, S., Brown, D.A., Salis, A., and Breit, S.N. (2016). Anorexia-cachexia and obesity treatment may be two sides of the same coin: role of the TGF-b superfamily cytokine MIC-1/GDF15. Int. J. Obes. *40*, 193–197.

Tsai, V.W.W., Husaini, Y., Sainsbury, A., Brown, D.A., and Breit, S.N. (2018). The MIC-1/GDF15-GFRAL pathway in energy homeostasis: implications for obesity, cachexia, and other associated diseases. Cell Metab. *28*, 353–368.

Unsicker, K., Spittau, B., and Krieglstein, K. (2013). The multiple facets of the TGF-β family cytokine growth/differentiation factor-15/macrophage inhibitory cytokine-1. Cytokine Growth Factor Rev. *24*, 373–384.

Vila, G., Riedl, M., Anderwald, C., Resl, M., Handisurya, A., Clodi, M., Prager, G., Ludvik, B., Krebs, M., and Luger, A. (2011). The relationship between insulin resistance and the cardiovascular biomarker growth differentiation factor-15 in obese patients. Clin. Chem. *57*, 309–316.

Wang, X., Baek, S.J., and Eling, T.E. (2013). The diverse roles of nonsteroidal anti-inflammatory drug activated gene (NAG-1/GDF15) in cancer. Biochem. Pharmacol. *85*, 597–606.

Welsh, J.B., Sapinoso, L.M., Kern, S.G., Brown, D.A., Liu, T., Bauskin, A.R., Ward, R.L., Hawkins, N.J., Quinn, D.I., Russell, P.J., et al. (2003). Large-scale delineation of secreted protein biomarkers overexpressed in cancer tissue and serum. Proc. Natl. Acad. Sci. USA *100*, 3410–3415.

Westerterp, K.R. (1999). Obesity and physical activity. Int. J. Obes. Relat. Metab. Disord. *23* (*Suppl 1*), 59–64.

Wollert, K.C., Kempf, T., and Wallentin, L. (2017). Growth differentiation factor 15 as a biomarker in cardiovascular disease. Clin. Chem. *63*, 140–151.

Wu, Q., Jiang, D., and Chu, H.W. (2012). Cigarette smoke induces growth differentiation factor 15 production in human lung epithelial cells: implication in mucin over-expression. Innate Immun. *18*, 617–626.

Xiong, Y., Walker, K., Min, X., Hale, C., Tran, T., Komorowski, R., Yang, J., Davda, J., Nuanmanee, N., Kemp, D., et al. (2017). Long-acting MIC-1/GDF15 molecules to treat obesity: Evidence from mice to monkeys. Sci. Transl. Med. *9*, 1–12.

Yang, H., Park, S.H., Choi, H.J., and Moon, Y. (2010). The integrated stress response-associated signals modulates intestinal tumor cell growth by NSAID-activated gene 1 (NAG-1/MIC-1/PTGF-β). Carcinogenesis *31*, 703–711.

Yang, L., Chang, C.C., Sun, Z., Madsen, D., Zhu, H., Padkjær, S.B., Wu, X., Huang, T., Hultman, K., Paulsen, S.J., et al. (2017). GFRAL is the receptor for GDF15 and is required for the anti-obesity effects of the ligand. Nat. Med. *23*, 1158–1166.

Yokoyama-Kobayashi, M., Saeki, M., Sekine, S., and Kato, S. (1997). Human cDNA encoding a novel TGF-beta superfamily protein highly expressed in placenta. J. Biochem. *122*, 622–626.

STAR★METHODS

KEY RESOURCES TABLE

REAGENT or RESOURCE	SOURCE	IDENTIFIER
Antibodies		
GDF15 (G-5)	Santa Cruz	Cat# sc-377195
GADD 153 (B-3)	Santa Cruz	Cat# sc-7351; RRID: AB_627411
Phospho S51 EIF2a	Epitomics/Abcam	Cat# ab32157; RRID: AB_732117
ATF4	Dr David Ron (CIMR)	(Harding et al., 2000a)
Calnexin	Abcam	Cat# ab75801; RRID: AB_1310022
Anti-rabbit IgG, HRP-linked Antibody	Cell Signaling Technologies	Cat# 7074; RRID: AB_2099233
Anti-mouse IgG, HRP-linked Antibody	Cell Signaling Technologies	Cat# 7076; RRID: AB_330924
Biological Samples		
C57BL/6J mice tissues	Charles River	JAX C57BL/6J; RRID: IMSR_JAX:000664
C57BL/6J mice tissues	In house (University of Cambridge)	N/A; RRID: IMSR_JAX:000664
Human samples (Blood)	Various	See Method Details for details
Chemicals, Peptides, and Recombinant Proteins		
Recombinant Human GDF15	Peprotech	Cat# 120-28
Lithium Chloride	Sigma	Cat# LX0331
Tunicamycin	Sigma	Cat# T7765
Thapsigargin	Sigma	Cat# T9033
Cobalt(II) chloride hexahydrate	Sigma	Cat# C8661
L-Histidinol dihydrochloride	Sigma	Cat# H6647
ISRIB	Sigma	Cat# SML0843
PERKi - GSK2606414	Calbiochem	Cat# 516535
TRI Reagent (Triazol)	Sigma	Cat# T9424
Chloroform	Honeywell	Cat# C2432
Ethanol, puriss. p.a., absolute, $\geq$ 99.8% (GC)	Sigma	Cat# 32221-M
DMEM	Sigma	Cat# D6546
D-PBS	Sigma	Cat# D8537
Hanks' Balanced Salt Solution	Sigma	Cat# H9269
L-Glutamine	Sigma	Cat# G7513
Penicillin-Streptomycin	Sigma	Cat# P0781
MEM Non-essential Amino Acid	Sigma	Cat# M7145
Sodium pyruvate	Sigma	Cat# S8636
2-Mercaptoethanol	GIBCO	Cat# 31350-010
FBS	GIBCO	Cat# 10270-106
FetalClone II Serum	Hyclone	Cat# SH30066.03
Sybr Green MasterMix	Applied Biosystems	Cat# 4309155
Taqman MasterMix	Applied Biosystems	Cat# 4304437
cOmplete, Mini Protease Inhibitor Cocktail	Sigma	Cat# 11836153001
PhosSTOP	Sigma	Cat# 4906845001
Random primers	Promega	Cat# C1181
RNasin Plus Ribonuclease inhibitor	Promega	Cat# N2611
MMLV Reverse Transcriptase	Promega	Cat# M1701
DNase1	QIAGEN	Cat# 79254
DNase1	Thermofisher Scientific	Cat# 18068015
Lipofectamine RNAiMAX	Invitrogen	Cat# 13778-150

(*Continued on next page*)

Continued

REAGENT or RESOURCE	SOURCE	IDENTIFIER
Collagenase Type II from Clostridium histolyticum	Sigma	Cat# C6885
NuPAGE 4-12% Bis-Tris Protein Gels, 1.5 mm, 10-well	Novex	Cat# NP0335BOX
NuPAGE MOPS SDS Running Buffer (20X)	Novex	Cat# NP0001
NuPAGE LDS Sample Buffer (4X)	Novex	Cat# NP0007
iBlot Transfer Stack, nitrocellulose, regular size	Invitrogen	Cat# IB301001
Dual Color Standards	BIO-RAD	Cat# 1610374
Bovine Serum Albumin	Sigma	Cat# A7906
Immobilon Western (Chemiluminescent HRP Substrate)	Millipore	Cat# WBKLS0500
ECL Western Blotting Detection Reagents	GE Healthcare	Cat# RPN2106
Dolethal 200mg/mL Solution for injection	Vetoquinol UK Ltd	N/A
Critical Commercial Assays		
RNAeasy Mini Kit	QIAGEN	Cat# 74106
Qiashredder	QIAGEN	Cat# 79656
Human total GLP-1	Meso Scale Discovery	Cat# K150JVC-1
Human Active GLP-1	R&D Systems	Cat# DY957
Human Insulin	Diasorin	Cat# 310360
Human GDF15 Elisa	R&D Systems	Cat# DY957
Human FGF21 Elisa	R&D Systems	Cat# DF2100
Human Leptin Elisa	In-house platform system using R&D Leptin: Cat # MAB398, BAM398 and 398-LP	https://www.cuh.nhs.uk/core-biochemical-assay-laboratory
Mouse GDF15 Elisa	R&D Systems	Cat# DY6385
Mouse FGF21 Elisa	R&D Systems	Cat# MF2100
Mouse Leptin/insulin 2-Plex	Meso Scale Discovery	Cat# K15124C
Experimental Models: Cell Lines		
Mouse Embryonic Fibroblasts (MEFs)	Dr David Ron (CIMR)	(Scheuner et al., 2001; Harding et al., 2003)
HeLa	ATCC	Cat# CCL-2; RRID: CVCL_0030
HuH7	Dr Albert Pol, IDIBAPS, Barcelona	Cat# JCRB0403; RRID: CVCL_0336
A549	ATCC	Cat# CCL-185; RRID: CVCL_0023
3T3-L1	Zenbio	Cat# SP-L1-F
Experimental Models: Organisms/Strains		
M. musculus: C57BL/6J mice strain	Charles River	JAX C57BL/6J; RRID: IMSR_JAX:000664
M. musculus: C57BL/6J mice strain	In House (University of Cambridge)	N/A; RRID: IMSR_JAX:000664
M. musculus: C57BL/6N mice strain	Taconic	C57BL/6NTac; RRID: MGI:5658006
Oligonucleotides		
Control siRNA	Dharmacon	Cat# D-001810-10-20
DDIT3 siRNA smartpool On-target Plus	Dharmacon	Cat# L-062068-00-0005
Human GDF15 Taqman assay	Thermo Fisher Scientific	Cat# Hs00171132_m1
Human FGF21 Taqman assay	Thermo Fisher Scientific	Cat# Hs00173927_m1
Mouse GDF15 Taqman assay	Thermo Fisher Scientific	Cat# Mm00442228_m1
Mouse FGF21 Taqman assay	Thermo Fisher Scientific	Cat# Mm00840165_g1
Human GAPDH Taqman assay	Thermo Fisher Scientific	Cat# Hs02758991_g1
Human HPRT Taqman assay	Thermo Fisher Scientific	Cat# Hs02800695_m1
Mouse HPRT Forward (AGCCTAAGATGAGCGCAAGT); Mouse HPRT Reverse (GGCCACAGGACTAGAACACC)	This paper	N/A

(*Continued on next page*)

Continued

REAGENT or RESOURCE	SOURCE	IDENTIFIER
Mouse B2M Forward (ACTGATACATACGCCTGCAGAGTT); Mouse B2M Reverse (TCACATGTCTCGATCCCAGTAGA)	This paper	N/A
Mouse 36b4 Forward (AGATGCAGCAGATCCGCAT); Mouse 36b4 Reverse (GTTCTTGCCCATCAGCACC)	This paper	N/A
Mouse F4/80 Forward CAGATACAGCAATGCCAAGCA; Mouse F4/80 Reverse GATTGTGAAGGTAGCATTCACAAGTG; Mouse F4/80 Probe FAM- GCAGGGCAGGGATCTTGGTTATGC-TAMRA	This paper	N/A
Mouse CHOP Forward (CCACCACACCTGAAAGCAGAA); Mouse CHOP Reverse (AGGTGAAAGGCAGGGACTCA)	This paper	(Oslowski and Urano, 2011)
Mouse ATF4 Forward (GGGTTCTGTCTTCCACTCCA); Mouse ATF4 Reverse (AAGCAGCAGAGTCAGGCTTTC)	This paper	(Oslowski and Urano, 2011)
Software and Algorithms		
GraphPad PRISM 7	1992-2017 GraphPad Software	RRID: SCR_000306
Illustrator (CS6)	Adobe	RRID: SCR_010279
Photoshop (CS6)	Adobe	RRID: SCR_014199
Other		
Chow diet (mouse studies)	Safe Diets	Cat# R105-25
Chow diet (mouse studies)	Purina (Lab Diet)	Cat# 5053
45% High Fat Diet (mouse studies)	Research Diets	Cat# D12451i
E Ensure Plus	Abbot Laboratories	N/A
ImageQuant LAS 4000	GE Healthcare	Cat# 28955810
Nanodrop 2000	Thermofisher Scientific	N/A
FastPrep-24	MP Biomedical	Cat# 116004500
AlphaTrack2 Glucometer	Abbot Laboratories	Cat# CFMU305-H0201
AlphaTrack2 strips	Zoetis	Cat# 71681-01
Hematocrit tubes Na-Heparinized	Hawksley	Cat# 01605-00
Microtube 1.1 mL Z Gel	Sarstedt AG & Co	Cat# 41.1378.005
Microtube 1.1 mL Z Gel	Sarstedt AG & Co	Cat# 41.1500.005
Lysing Matrix D, 2 mL Tube	MP Biomedical	Cat# 116913100
Sterile Cell strainer (100 μm nylon mesh)	Fisherbrand	22363549
Minispec LF series (TD-NMR)	Bruker	Cat# LF50
Lunar PIXImus Mouse Densitometer	GE Healthcare Systems	N/A
iBlot Dry Blotting System	Invitrogen	N/A
PowerPac Universal Power Supply	BIO-RAD	Cat# 1645070
XCell SureLock Mini-Cell Electrophoresis System	Invitrogen	Cat# EI0001
QuantStudio 7 Flex Real-Time PCR System	ThermoFisher Scientific	Cat# 4485701

CONTACT FOR REAGENT AND RESOURCE SHARING

Further information and requests for resources and reagents should be directed to and will be fulfilled by the Lead Contact, David Savage (dbs23@medschl.cam.ac.uk).

EXPERIMENTAL MODEL AND SUBJECT DETAILS

Human Subjects

Human Study 1: Oral Glucose Tolerance Test (OGTT)/Mixed Meal

The liquid meal test included 12 healthy adult volunteers (3 male/9 female) with a mean ± SD age and BMI of 28 ± 9 years and 23.14 ± 2.74 kg/m^2 respectively. Six healthy adult volunteers (4 male/2 female) were recruited to participate in the oral glucose tolerance test study with mean ± SD age of 30 ± 8 years and BMI of 25.05 ± 3.73 kg/m^2. Both studies were completed at the NIHR Wellcome Trust Clinical Research Facility and the Wellcome-MRC Translational Research Facility, Cambridge Biomedical Campus, UK. Ethical approval was obtained from the East of England Cambridge South research ethics committee Ref: 16/EE/0338 and the East of

England Cambridgeshire and Hertfordshire research ethics committee Ref: 013/EE/0195. Participants provided written consent prior to participation in the study.

Human Study 2: 48 Hours of Caloric Restriction

14 healthy male volunteers were recruited as participants in the study. The mean ± SD age and BMI of participants was 23.53 ± 2.70 years and 22.08 ± 2.0 kg/m^2 respectively. Ethical approval was obtained from the Cambridge local research ethics committee (Ref: 13EE0107). All participants provided written informed consent before taking part in the study which was completed at the NIHR Wellcome Trust Clinical Research Facility, Cambridge Biomedical Campus, UK.

Human Study 3: Low Calorie Diet Intervention

50 overweight (BMI > 27 and < 30 kg/m^2) or obese (BMI 30-40 kg/m^2) participants were enrolled in this study. 33 (3 male and 30 female) were included in this analysis after accounting for withdrawals and insufficient biological samples. Mean ± SD age and BMI were 38.8 ± 8.8 years and 35.1 ± 3.1 kg/m^2 respectively. The study protocol was approved by the Florida Hospital Institutional Review Board and was carried out in accordance with the Declaration of Helsinki. Clinical trial number: NCT01616082 (https://clinicaltrials.gov). Before taking part in the study, all participants were evaluated for eligibility. All participants provided their written consent to take part in the study.

Human Study 4: Calorie Restriction

In total 13 healthy adult participants (7 male/6 female) were recruited to the study. The inclusion criteria for participation were age 18-45 years, percent body fat from DEXA > 12% for males and > 15% for females. Mean ± SD age of participants was 29.7 ± 6.1 years and BMI was 25.04 ± 3.32 kg/m^2. The study protocol was initially reviewed by the Regional Ethics Committee of Norway (2017/1052; REK sør-øst B) with the decision that the research project was outside the Act on Medical Health Research, confirmed in a letter of exemption (2017/1052b). The study was then approved by the Ethics Committee at the Norwegian School of Sport Sciences (15-220817). The study was undertaken at the Norwegian School of Sports Sciences, Oslo, Norway. Written consent was obtained from volunteers prior to participation in the study.

Human Study 5: 7 Day High Fat Diet Overfeeding

A total of 28 adult participants (25 males/3 females) were included in the study. Mean ± SD age and BMI of the study cohort were 22.6 ± 3.7 years and 24.2 ± 2.5 kg/m^2 respectively. Subjects were physically active (taking part in at least 3 × 30 min of moderate-intensity physical activity each week), non-smokers, with no diagnosis of cardiovascular or metabolic disease, not taking any medication, and body mass was stable for at least 3 months. Both studies were approved by the Loughborough University Ethical Subcommittee for Human Participants (R13-P171 and R16-P132). All participants gave written consent to take part after the experimental procedures.

Human Study 6: 8 Week Overfeeding

20 healthy adult volunteers (11 male /9 female) with a mean ± SD age and BMI of 24.3 ± 4.3 years and 25.2 ± 3.0 kg/m^2 respectively who led a sedentary lifestyle (less than 2 h of moderate to vigorous exercise per week) were recruited to an 8 week overfeeding study. Written consent approved by the Pennington Biomedical Research Center Institutional Review Board was provided by all participants. This trial was registered at clinicaltrials.gov (number NCT00565149). The study was undertaken at the Pennington Biomedical Research Center (Baton Rouge, Louisiana, USA).

Mouse Studies

Mouse Study 1: Fed/Fast Study

Ten C57BL/6J mice were purchased from Charles River (Charles River Ltd, Manston Rd, Margate, Kent, CT9 4LT) at 7-8 weeks of age. Mice were maintained in open vented cages with group housing (2 or 3 per cage) in a 12 h light/12 h dark cycle (lights on 07:00–19:00), temperature-controlled (22°C) facility, with *ad libitum* access to food and water. This research was regulated under the Animals (Scientific Procedures) Act 1986 Amendment Regulations 2012 following ethical review by the University of Cambridge Animal Welfare and Ethical Review Body (AWERB).

Mouse Study 2 and 3: Short- and Long- Term High Fat Diet Studies

C57BL/6J mice were purchased from Charles River (Charles River Ltd, Manston Rd, Margate, Kent, CT9 4LT) or bred in-house for some long-term HFD experiments. Mice were maintained in ventilated cages with group housing (2 or 3 per cage) in a 12 h light/12 h dark cycle (lights on 06:00–18:00), temperature-controlled (20-24°C) facility, with *ad libitum* access to food and water. All mice were fed either *ad libitum* or as stated otherwise prior to harvesting tissue and serum analysis. This research was regulated under the Animals (Scientific Procedures) Act 1986 Amendment Regulations 2012 following ethical review by the University of Cambridge Animal Welfare and Ethical Review Body (AWERB).

Mouse Study 4: Lysine Nutritional Deficiency Experiment

Mice were originally purchased at Janvier Labs (Route du Genest, 53940 Le Genest-Saint-Isle, France) and bred in-house. All mice were maintained in standard housing conditions (22°C) on a 12 h light-dark cycle (lights on 08:00-20:00). Animal experiments were carried out in accordance with INRA guidelines in compliance with European animal welfare regulation. Mouse maintenance and all experiments have been approved by the institutional animal care and use committee, in conformance with French and European Union laws (permission to experiment on mice #5558, animal facilities agreement D6334515, GMO agreement #4713).

For the lysine nutritional deficiency experiment, an experimental diet was manufactured in the INRA diets core facility (Unité de Préparation des Aliments Expérimentaux, INRA) and nutritional experiments were performed as previously described

(Chaveroux et al., 2016; Maurin et al., 2005). Briefly, the nutritional deficiency in an essential amino acid is carried out by means of experimental diets in which the protein fraction is replaced by a mixture of free amino acids.

Mouse Study 5: Conditioned Taste Aversion (CTA) Test

Male C57BL/6N mice were obtained from Taconic Farms (25-30 g). All mice were maintained in standard housing conditions (21-24°C; 45% humidity) on a 12 h light-dark cycle (lights on 06:00, lights off 18:00). Mice were singly housed in the BioDAQ caging system (Research Diets, New Brunswick, NJ, USA) and allowed *ad libitum* access to tap water and standard rodent chow (Purina 5053) unless otherwise noted. All procedures were approved by the Pfizer-Massachusetts Animal Care and Use Committee.

Eukaryotic Cell Lines

Mouse embryonic fibroblast (MEF) cells lines were obtained from David Ron (CIMR/IMS, Cambridge) and maintained in Dulbecco's Modified Eagle's Medium supplemented with 10% (vol/vol) fetal bovine serum (FBS), 2 mM L-glutamine, penicillin/streptomycin, 1% Sodium Pyruvate, 1% Non-Essential Amino Acids and 2-Mercaptoethanol. HeLa (human cervical carcinoma obtained from ATCC), HuH7 (human hepatocarcinoma obtained from Albert Pol, IDIBAPS, Barcelona), A549 (human lung epithelial carcinoma obtained from ATCC) were cultured in the same media as MEFs but without 2-Mercaptoethanol. 3T3-L1 preadipocytes (obtained from Zenbio) cells were cultured in complete DMEM containing 10% newborn calf serum (NCS), 2 mM L-Glutamine, Penicillin-Streptomycin, 1% Non-Essential Amino Acids and 1% Sodium Pyruvate. All cells were maintained at 37°C in a humidified atmosphere of 5% CO2

METHOD DETAILS

Human Studies

Human Study 1: Oral Glucose Tolerance Test (OGTT)/Mixed Meal

On the day before the assessment all participants received a standardized evening meal at 18:00, before commencing an overnight fast. The energy content of the meal was one third of a participant's daily requirements estimated from predicted resting metabolic rate and multiplied by an activity factor of 1.35 (Schofield, 1985; Westerterp, 1999). Meal composition consisted of 30%–35% fat, 12%–15% protein and 50%–55% carbohydrate by energy. Anthropometric measurements were acquired for all participants on arrival to the clinical research facility. Participants were cannulated prior to administration of an oral liquid meal consisting of a 200 mL Ensure Plus (Total energy 330 kcal; Protein 16.7%, Carbohydrate 53.8%, Fat 29.5%) or a glucose drink (50 g anhydrous glucose in 200 mL water) with these particular participants described in Roberts et al. (2018). Blood samples were taken at 30 min intervals over the 180 min duration of the study. EDTA and Lithium heparin samples were placed immediately on ice while serum samples remained at room temperature for 30 min prior to centrifugation at 4°C at 3500 rpm for 10 min, plasma was frozen on dry ice and stored at −70°C until the time of biochemical analysis. Assays were completed by the Cambridge Biochemical Assay Laboratory, University of Cambridge. Serum GDF15 measurements were undertaken with antibodies & standards from R&D Systems (R&D Systems Europe, Abingdon UK) using a microtiter plate-based two-site electrochemiluminescence immunoassay using the MesoScale Discovery assay platform (MSD, Rockville, Maryland, USA). Plasma glucose was determined using a hexokinase assay on a Siemens Dimension ExL Analyzer. Plasma insulin measurements using the Diasorin Liaison XL automated onestep chemiluminesence immunoassay (Diasorin S.p.A, 13040 Saluggia (VC), Italy). Plasma total GLP-1 was measured by microtiter plate-based two-site electrochemiluminescence immunoassay using a Meso Scale Discovery kit (Gaithersburg, MD, USA). FGF21 levels were measured in duplicate on serum samples using the human FGF21 Quantikine ELISA kit (R&D Systems).

Human Study 2: 48 Hours of Caloric Restriction

Study volunteers attended the clinical research facility after an overnight fast. During the study, participants were required to eat all of the meals provided by the research team. For the first 24 h of the study (day 1), all of the meals provided to participants contained 100% of their estimated daily energy requirements based on the Scholfield equation and were composed of 50% carbohydrate, 30% fat and 20% protein (Schofield, 1985). Baseline blood tests were acquired upon waking on day 2 of the study. For the following 48 h (day 2 and 3) participants were calorie restricted to meals containing 10% of their daily estimated energy requirements. Blood sampling was repeated upon waking on day 4 of the study protocol, prior to refeeding. Serum samples remained at room temperature for 30 min prior to centrifugation at 4°C at 3500 rpm for 10 min, plasma was frozen on dry ice and stored at −80°C until the time of biochemical analysis. Assays were completed by the Cambridge Biochemical Assay Laboratory, University of Cambridge. Serum GDF15 measurement were undertaken with antibodies & standards from R&D Systems (R&D Systems Europe, Abingdon UK) using a microtiter plate-based two-site electrochemiluminescence immunoassay using the MesoScale Discovery assay platform (MSD, Rockville, Maryland, USA).

Human Study 3: Low Calorie Diet Intervention

At day 0, prior to initiation of the low-calorie dietary (LCD) intervention, baseline fasting blood sampling and anthropometry were measured. Participants were provided with dietary counselling and meal-replacement shakes at this and subsequent visits, and the LCD was initiated. Participants received a low-calorie diet for 8 weeks at approximately 1000 kcal per day, replacing 2 meals with approximately 600 kcal of meal replacement shakes followed by a dinner of approximately 400 kcal. Dinners were chosen from an approved list of Lean Cuisine and Healthy Choice brand meals. Participants were free living for the duration of the study and attended at days 14 and 28 of the intervention for assessment where blood sampling and anthropometric measures were repeated.

EDTA plasma samples underwent centrifugation at 4°C, 4000 rpm for 15 min and stored at −80°C until analysis. Biochemical assays were undertaken at the Translational Research Institute for Metabolism and Diabetes (Florida Hospital). Plasma GDF15 was measured using antibodies & standards from R&D Systems (R&D Systems Europe, Abingdon UK) using a microtiter plate-based two-site electrochemiluminescence immunoassay using the MesoScale Discovery assay platform (MSD, Rockville, Maryland, USA). Plasma leptin was assayed using the MesoScale Discovery platform, (Human Leptin).

Human Study 4: Calorie Restriction

Participants were free living for the duration of the caloric restriction. Anthropometric measurements were acquired at baseline and at the end of the study. On day 0 all participants had a breakfast meal *ad libitum* prior to commencing the caloric restriction to 0 kcal per day for a total of 7 days. Water was permitted throughout the study. During the study, weight (mean ± SD) fell from 79.6 ± 5.0 kg at baseline to 73.8 ± 4.8 kg after 1 week of fasting. The measurements of mean ± SD body fat were acquired by dual-energy X-ray absorptiometry (DEXA) and are reported as an average of two measurements at baseline on day −1 and 0 (18.6 ± 1.8 kg) or following the fast on days 6 and 7 (17.3 ± 1.9 kg). Participants attended the research facility each morning for phlebotomy where both serum and plasma (EDTA and Lithium Heparin) samples were acquired. Plasma samples were immediately placed on ice while serum samples remained at room temperature for 30 min to coagulate prior to centrifugation at 4°C at 3500 rpm for 10 min. Samples were then immediately frozen on dry ice and stored at −80°C until the time of biochemical analysis. Plasma Leptin and GDF15 measurements were undertaken at the Cambridge Biochemical Assay Laboratory, University of Cambridge using antibodies & standards from R&D Systems (R&D Systems Europe, Abingdon UK). A two-site microtiter plate-based Delfia assay measured Leptin. GDF15 was measured using a microtiter plate-based two-site electrochemiluminescence immunoassay using the MesoScale Discovery assay platform (MSD, Rockville, Maryland, USA). The analytic processes of β-Hydroxybutyrate were conducted according to the manufacturer's instructions. Plasma concentration of β-Hydroxybutyrate (mmol/l) were undertaken at the Department of Clinical Medicine, Diabetes and Hormone Diseases - Medical Research Laboratory, Aarhus University, Denmark using a kinetic enzymatic method, based on the oxidation of D-3-hydroxybytyrate to acetoacetate by the enzyme 3-Hydroxybutyrate dehydrogenase (Randox Laboratories Ltd., Crumlin, UK) and measured on the Cobas c111 system (Roche Diagnostics International Ltd, Rotkreuz, Switzerland). FGF21 levels were measured in duplicate on serum samples using the human FGF21 Quantikine ELISA kit (R&D Systems).

Human Study 5: 7 Day High Fat Diet Overfeeding

Prior to the start of the study, subjects attended the research facility at Loughborough University for an initial assessment of their baseline anthropometric characteristics. This information was then used to estimate resting energy expenditure (REE) using validated equations (Mifflin et al., 1990). A standard correction for physical activity (1.6 and 1.7 times REE for females and males, respectively) was applied in order to estimate total daily energy requirements. This information was then used to determine individual energy intakes for the overfeeding period. Experimental trials were conducted immediately before and after 7 days of high-fat overfeeding. Briefly, subjects arrived at the laboratory in the morning (07:00-09:00) after an overnight fast (≥10 h), having refrained from strenuous exercise for 48 h and having avoiding alcohol or caffeine intake for 24 h. Body mass was recorded after subjects had voided. A venous blood sample was then obtained after 30 min of seated rest. Blood samples were collected for plasma (EDTA) or serum. Blood samples were then centrifuged, and the resulting plasma / serum stored at −20°C until analysis. Upon completion of the first experimental trial, subjects were provided with all food to be consumed for the following 7 days. The high-fat diet provided 19974 ± 474 kJ per day (48 ± 1% greater than estimated daily requirement), with 178 ± 5 g [15%] protein, 245 ± 5 g [21%] carbohydrate, and 342 ± 9 g [64%] fat intake. Diet compliance was assessed by daily interview. Plasma glucose concentration was determined using a spectrophotometric assay (Glucose PAP; Horiba Medical, Northampton, UK) and semi-automatic analyzer (Pentra 400; Horiba Medical, Northampton, UK). Serum insulin concentration was determined by ELISA (EIA-2935; DRG Instruments GmBH, Marburg, Germany). Serum Leptin and GDF15 measurements were undertaken at the Cambridge Biochemical Assay Laboratory, University of Cambridge using antibodies & standards from R&D Systems (R&D Systems Europe, Abingdon UK). A two-site microtiter plate-based Delfia assay was used to measure Leptin. GDF15 was measured using a microtiter plate-based two-site electrochemiluminescence immunoassay using the MesoScale Discovery assay platform (MSD, Rockville, Maryland, USA). FGF21 levels were measured in duplicate on serum samples using the human FGF21 Quantikine ELISA kit (R&D Systems).

Human Study 6: 8-Week Overfeeding

Details of this study were previously described (Bray et al., 2012, 2015). Briefly, this was a randomized, parallel-arm, in-patient study. Participants remained in-patients at the Biomedical Research Center for approximately 12 weeks without leaving. The first 13–25 days of the in-patient stay (Baseline) were used to establish energy requirements for weight maintenance. The baseline diet consisted of 361 g of carbohydrates, 67 g of fat, 90 g protein for a total of 2412 kcal. Once weight stability was achieved, baseline assessments were performed, including blood draws and measurements of body composition by dual-energy X-ray absorptiometry (DEXA). Overfeeding was planned at approximately 40% above energy requirements for weight maintenance or approximately 1000 kcal/d (4180 kJ/d). During the final 24 h period, the diet was returned to the baseline components and the same baseline assessments were performed at the end of the 8 week overfeeding. Participants ate all food provided during the study period. Plasma glucose was measured using a glucose oxidase electrode (DXC 600 Pro; Beckman Coulter), and insulin was measured by an immunoassay (Immulite 2000; Siemens). Plasma free fatty acids (FFAs) were measured with a high-sensitivity Wako kit. Plasma GDF15 measurements were undertaken with antibodies & standards from R&D Systems (R&D Systems Europe, Abingdon UK) using a microtiter plate-based two-site electrochemiluminescence immunoassay using the MesoScale Discovery assay platform (MSD, Rockville, Maryland, USA).

Mouse Studies

Mouse Study 1: Fed/Fast Study

When aged 11-12 weeks old, on day 1 of the study the mice were divided into two groups of five mice, either “fed” or “fasted.” The body weight of the two groups at study start were matched (fed versus fast, mean ± SEM; 27.68 ± 0.45 g versus 27.70 ± 0.59 g). At 09:00, mice were transferred into clean cages in the same grouped arrangement as during the maintenance period. Home cage bedding was also transferred into clean cages to reduce stress. Animals in the “fasted” group had all food removed, animals in the “fed” had *ad libitum* access to food. All animals had free access to water. 24 h later (09:00 on day 2 of study) mice were weighed then received a terminal dose of anesthetic (Dolethal 200 mg/mL solution, Vetoquinol UK Ltd.) given via the intraperitoneal route. Once unresponsive, blood was collected by cardiac puncture, transferred into a Microtube 1.1 mL Z-Gel (Sarstedt AG & Co) and spun at 10 000 x g for 5 min at room temperature. Serum was collected, frozen on dry ice and stored at −80°C until analyzed. After cardiac puncture, body composition was measured using DEXA with a Lunar PIXImus Mouse Densitometer (GE Healthcare Systems) and tissue was harvested, frozen on dry ice and stored at −80°C until being processed.

Mouse Study 2 and 3: Short- and Long- Term High Fat Diet Studies

For the short-term high fat diet study (Mouse Study 2), 17-18 week old mice were fed a 45% high-fat diet (D12451i, 45% kcal as fat, 4.7 kcal/g, Research Diets) for 1, 3 or 7 days. A separate control group was kept on a chow diet (Safe Diets, DS-105). On the morning (10:00) of the specified days, mice were weighed and blood collected by cardiac puncture into microtubes containing serum gel with clotting activator and centrifuged at 13 000 rpm for 10 min at 4°C and stored at −80°C for serum GDF15 and insulin measurements. Mouse glucose levels were measured from approximately 2 μl blood drops using a glucometer (AlphaTrak2; Abbot Laboratories) and glucose strips (AlphaTrak2 test 2 strips, Abbot Laboratories, Zoetis). Tissues were harvested and weighed.

For the long-term chronic high fat diet study (Mouse Study 3), 9 week-old male mice were subjected to either a chow or high fat diet (as in short-term HFD) over a period of 18 weeks. All mice were weighed weekly and body composition was determined every 4 weeks by Time-Domain Nuclear Magnetic Resonance (TD-NMR) using a Minispec Live Mice Analyzer (LF50, Bruker). Tail blood samples were collected into heparinized micro blood tubes (01605-00, Hawksley), centrifuged at 13,000 x g for 4 min for plasma GDF15, leptin and insulin measurement. Mouse glucose levels were measured as described above in the short-term HFD study. At the end of the experiment, mice were fasted for 4 h and tissue was harvested and stored at −80°C. For isolating stromo-vascular and adipocyte fractions, epididymal adipose tissue was minced into small pieces and resuspended in 5 mL Hanks’ Balanced Salt Solution (Sigma) containing collagenase Type I (Sigma). The tissue was completely disaggregated by incubation in a 37°C shaker for approximately 10 min. The digested material was filtered through a 100 μm nylon mesh cell strainer, and 10 mL of 10% FBS DMEM added. After a 5-10 min incubation at room temperature, the upper phase containing the adipocytes was transferred into a new tube. The remaining supernatant was centrifuged at 700 x g for 10 min and the pellet containing the stromo- vascular fraction was collected. Both fractions were frozen at −80°C until further analysis.

Mouse Study 4: Lysine Nutritional Deficiency Experiment

Eighteen 10 week old C57BL/6J female mice were habituated to the control experimental diet (containing 20 free amino acids) for one week. The night before the experiment, mice were fasted for 16 h before offering them a control meal or a meal lacking lysine. Mice were divided into three groups of six mice: the first group was fasted overnight; the second group was fasted overnight, then fed the control diet; the third group was fasted overnight, then fed the lysine devoid diet. A blood sample was collected from fed mice at 1 h after the beginning of the meal by sub-mandibular sampling. At the time of sacrifice (4 h after the beginning of the meal for fed mice), the blood of all mice was withdrawn by cardiac puncture and treated with EDTA (500 mM) along with the tissues being harvested and stored at −80°C for analysis.

Mouse Study 5: Conditioned Taste Aversion (CTA) Test

Human recombinant GDF15 (Peprotech) was prepared in saline, which was used as the vehicle control. GDF15 was administered via subcutaneous (SC) injection as a single dose in all mouse studies. LiCl (Sigma) was also prepared in saline and administered via SC injection as a single dose in all mouse studies. Mice were acclimated (up to 3 days) to drinking from two water bottles to confirm lack of side preference prior to habituation. Mice were then habituated to overnight water restriction (days 1-3) followed by 1 h water bottle presentation (two bottles) and saline SC injection. On day 4 to begin conditioning, mice were instead given a novel 0.15% saccharin solution in both bottles instead of water for 1 h, followed by a SC injection of either saline, GDF15 (within the dose range that induces anorexia) or the positive control LiCl. Access to saccharin water was allowed for an additional 30 min and was then changed back to water until the next restriction. Day 5 was used as a GDF15 washout period using the days 1-3 bottle protocol. A second conditioning period was performed on day 6 followed by a washout period on day 7. On day 8, a standard two bottle preference test (saccharin versus water) was used to assess CTA development to the saccharin solution (1 h presentation after overnight water restriction). The CTA test was performed 48 hours after the last GDF15 injection, and volume measurements were for 1 hour. Fluid intake volume was calculated for both saccharin and water. The total volume drunk in saline group was 1.4 mL and there was no statistical differences in the treatment groups. Food weight was measured manually using a digital scale. Food weight was measured at 1 h and 4 h following a single injection of GDF15 given immediately prior to the onset of the dark cycle. A separate group of mice was used for plasma GDF15 pharmacokinetic analysis. Blood was collected at 0.25 h, 1 h, 4 h, 8 h, and 24 h after GDF15 injection in EDTA tubes containing AEBSF and aprotinin, and then centrifuged (10 000 rpm; 10 min) for plasma separation all at 4°C and then stored at −80°C. Plasma human GDF15 was measured using the human GDF15 Quantikine ELISA kit per manufacturer instructions.

Eukaryotic Cell Lines

Cells were seeded onto 6- or 12-well plates prior to stress treatments the following day for the times and concentrations indicated. Vehicle treatments (e.g., DMSO or ethanol) were used for control cells when appropriate.

siRNA Transfection and Knockdown CHOP

Wild-type MEFs were seeded onto 12-well plates and transfected with 30 nM control siRNA or siRNA for mouse CHOP using Lipofectamine RNAi MAX (Invitrogen) according to the manufacturer's instruction. 48 h post siRNA transfection, cells were treated with ISR stressors for 6 h and subsequently processed for RNA and protein expression analysis.

RNA Isolation/cDNA Synthesis/Q-PCR

Following treatments, cells were lysed with Buffer RLT (QIAGEN) containing 1% 2-Mercaptoethanol and processed through a Qiashredder with total RNA extracted using the RNeasy isolation kit according to manufacturer's instructions (QIAGEN). Meanwhile for mice, tissues were harvested and immediately snap frozen in liquid nitrogen and stored at −80°C until further analysis. For RNA isolation, approximately 30-50 mg of tissue was placed in Lysing Matrix D tubes and homogenized in 800 µl Triazol (Qiazol,QIAGEN) using the Fastprep-24 Homogenizer for 30 s at 4-6 m/s (MP Biomedical), or a rotor-stator homogenizer. The resultant supernatant was transferred to an RNase free tube and 200 µl chloroform (Sigma) added. The samples were vortexed and centrifuged at 13 000 rpm for 15 min at 4°C. The upper phase was then transferred to a RNase free tube and mixed with equal volume of 70% ethanol before loading onto RNA isolation spin columns (QIAGEN). RNA was then extracted (and in some instances treated with DNase1 on-column) using the RNeasy isolation kit following the manufacturer's instructions.

RNA concentration and quality was determined by Nanodrop. 400 ng - 500 ng of total RNA was treated with DNAase1 (Thermofisher Scientific) and then converted to cDNA using MMLV Reverse Transcriptase with random primers (Promega). Quantitative RT-PCR was carried out with either TaqMan Universal PCR Master Mix or SYBR Green PCR master mix on the QuantStudio 7 Flex Real time PCR system (Applied Biosystems). All reactions were carried out in either duplicate or triplicate and Ct values were obtained. Relative differences in the gene expression were normalized to expression levels of housekeeping genes, HPRT or GAPDH for cell analysis and to B2M and 36b4 geometrical mean for mouse data, using the standard curve method. Primer sequences are shown in the Key Resources Table.

Serum and Media Analysis

Tail blood samples from random fed or 4 h fasted (for the long term HFD study) mice were collected for serum analysis. Mouse leptin and insulin were measured simultaneously using a 2-plex Mouse Metabolic immunoassay kit from Meso Scale Discovery Kit (Rockville, MD, USA). The assay was performed according to the manufacturer's instructions and using calibrators provided by MSD. Mouse GDF15 was measured using a Mouse GDF15 DuoSet ELISA (R&D Systems) which had been modified to run as an electrochemiluminescence assay on the Meso Scale Discovery assay platform. Mouse FGF21 was analyzed by FGF21 Quantakine ELISA (R&D Systems) following the manufacturer's instructions. Mouse sample measurements were performed by the MRC MDU Mouse Biochemistry Laboratory [MRC_MC_UU_12012/5]. For the human studies, the details of the serum/plasma analysis performed are described separately for each study (see Method Details section of each study above). All FGF21 measurements were completed by the Cambridge Biochemical Assay Laboratory, University of Cambridge using the human FGF21 Quantikine ELISA kit (R&D Systems).

Immunoblotting

Following treatments, cells were washed twice with ice cold D-PBS and proteins harvested using RIPA buffer supplemented with cOmplete protease and PhosStop inhibitors (Sigma). The lysates were cleared by centrifugation at 13 000 rpm for 15 min at 4°C, and protein concentration determined by a Bio-Rad DC protein assay. Typically, 20-30 µg of protein lysates were denatured in NuPAGE 4 × LDS sample buffer and resolved on NuPage 4%–12% Bis-Tris gels (Invitrogen) and the proteins transferred by iBlot (Invitrogen) onto nitrocellulose membranes. The membranes were blocked with 5% nonfat dry milk or 5% BSA for 1 h at room temperature and incubated with the antibodies described in the STAR Methods table. Following a 16 h incubation at 4°C, all membranes were washed five times in Tris-buffered saline-0.1% Tween-20 prior to incubation with horseradish peroxidase (HRP)-conjugated anti-rabbit immunoglobulin G (IgG), HRP-conjugated anti-mouse IgG. The bands were visualized using Immobilon Western Chemiluminescent HRP Substrate (Millipore). All images were acquired on the ImageQuant LAS 4000 (GE Healthcare).

QUANTIFICATION AND STATISTICAL ANALYSIS

Quantitative data are reported as mean ± SD for cells and mean ± SEM for mouse data. As indicated in the figure legends, differences between means were assessed by two-tailed Student's t tests or One-way ANOVA or Two-way ANOVA with Bonferroni multiple comparisons test using either GraphPad Prism software (GraphPad, San Diego) or with SAS version 9.4, Cary, N. Carolina. Statistical significance was defined as $p < 0.05$.

Data from human studies was analyzed using GraphPad Prism software (GraphPad, San Diego). Parametric quantitative data is expressed as mean ± SEM and the difference in the mean was assessed using a Two tailed Student's t test. In the case of non-parametric data, it is reported as median (interquartile range) and compared using a Wilcoxon signed rank, Kruskal-Wallis or Mann Whitney test. Details of specific analyses are reported in the respective figure legends. Statistical significance was defined as $p < 0.05$.

Cell Metabolism, Volume *29*

Supplemental Information

GDF15 Provides an Endocrine Signal of Nutritional Stress in Mice and Humans

Satish Patel, Anna Alvarez-Guaita, Audrey Melvin, Debra Rimmington, Alessia Dattilo, Emily L. Miedzybrodzka, Irene Cimino, Anne-Catherine Maurin, Geoffrey P. Roberts, Claire L. Meek, Samuel Virtue, Lauren M. Sparks, Stephanie A. Parsons, Leanne M. Redman, George A. Bray, Alice P. Liou, Rachel M. Woods, Sion A. Parry, Per B. Jeppesen, Anders J. Kolnes, Heather P. Harding, David Ron, Antonio Vidal-Puig, Frank Reimann, Fiona M. Gribble, Carl J. Hulston, I. Sadaf Farooqi, Pierre Fafournoux, Steven R. Smith, Jorgen Jensen, Danna Breen, Zhidan Wu, Bei B. Zhang, Anthony P. Coll, David B. Savage, and Stephen O'Rahilly

Supplemental figures

GDF 15 provides an endocrine signal of nutritional stress in mice and humans

Satish Patel, Anna Alvarez-Guaita, Audrey Melvin, Debra Rimmington, Alessia Dattilo, Emily L. Miedzybrodzka, Irene Cimino, Anne-Catherine Maurin, Geoffrey P. Roberts, Claire L. Meek, Samuel Virtue, Lauren M. Sparks, Stephanie A. Parsons, Leanne M. Redman, George A. Bray, Alice P. Liou, Rachel M. Woods, Sion A. Parry, Per B. Jeppesen, Anders J. Kolnes , Heather P. Harding, David Ron, Antonio Vidal-Puig, Frank Reimann, Fiona M. Gribble, Carl J. Hulston, I. Sadaf Farooqi, Pierre Fafournoux[3], Steven R. Smith , Jorgen Jensen, Danna Breen, Zhidan Wu, Bei B. Zhang, Anthony P. Coll, David B. Savage, Stephen O'Rahilly

Figure S1

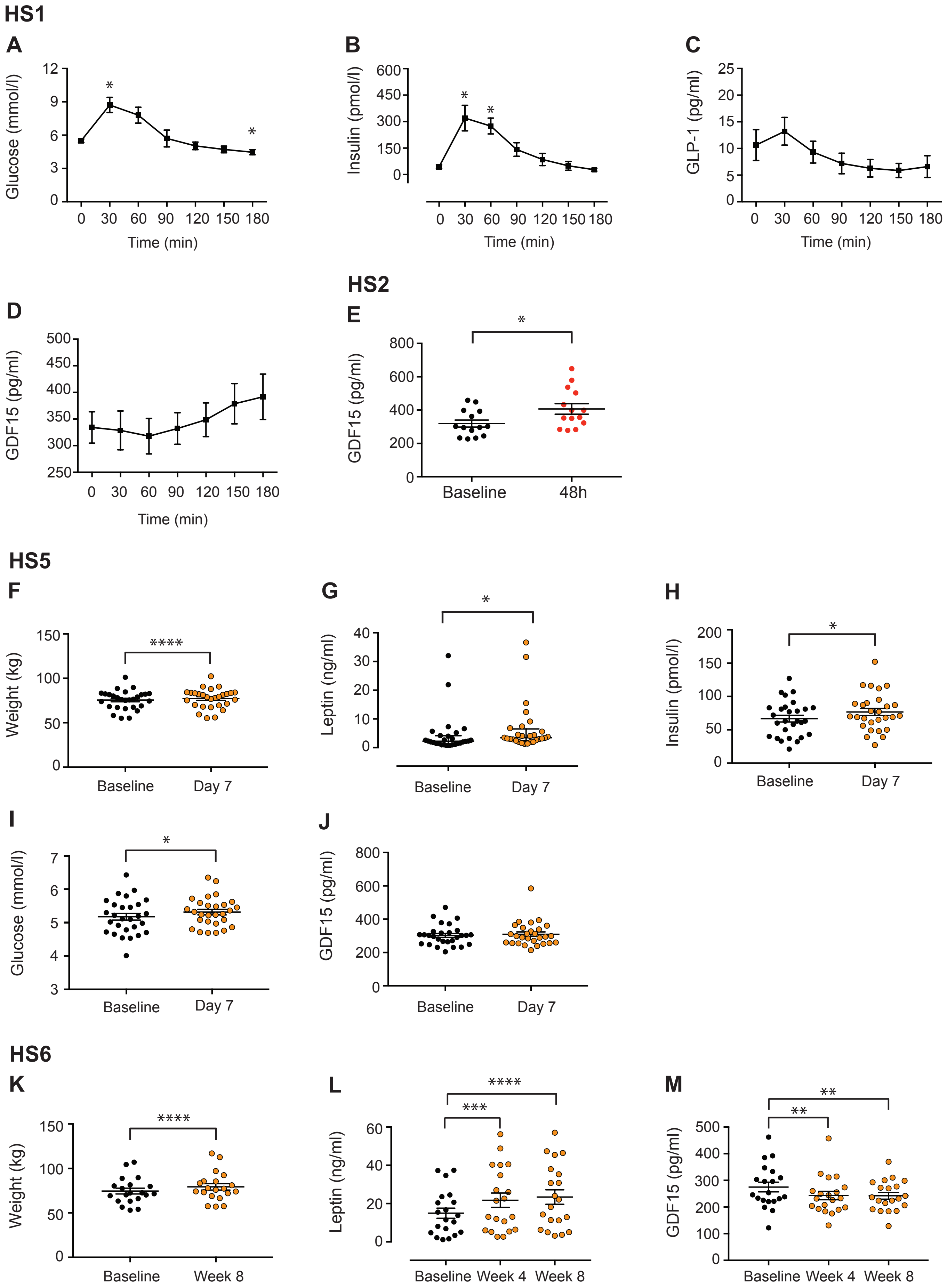
HS1
A
Glucose (mmol/l)
Time (min)
B
Insulin (pmol/l)
Time (min)
C
GLP-1 (pg/ml)
Time (min)
D
GDF15 (pg/ml)
Time (min)
HS2
E
GDF15 (pg/ml)
Baseline
48h
HS5
F
Weight (kg)
Baseline
Day 7
G
Leptin (ng/ml)
Baseline
Day 7
H
Insulin (pmol/l)
Baseline
Day 7
I
Glucose (mmol/l)
Baseline
Day 7
J
GDF15 (pg/ml)
Baseline
Day 7
HS6
K
Weight (kg)
Baseline
Week 8
L
Leptin (ng/ml)
Baseline
Week 4
Week 8
M
GDF15 (pg/ml)
Baseline
Week 4
Week 8

Supplemental Figure Legends

Figure S1 (related to Figure 1). **GDF15 levels in response to a meal or imposed caloric deficit in mice and humans.**

HS1-Human Study 1 (A-D): Plasma (A) glucose, (B) insulin, (C) GLP-1 and (D) GDF15 circulating levels in six healthy volunteers given an 50 g oral glucose tolerance test following an overnight fast. Blood samples were taken at serial intervals over the 180 min duration of the study. Data is expressed as mean ± SEM. * $p < 0.05$ comparing to Time 0 min by a One-way ANOVA with Bonferroni post-test.

HS2- Human study 2 (E) GDF15 levels of 14 healthy male volunteers undergoing 48 h of caloric restriction to 10 % of estimated energy requirement per day. Data is expressed as mean (SEM) and compared using a paired t-test, * $p < 0.05$.

HS5-Human study 5 (F-J): (F) Body weight, (G) leptin, (H) insulin, (I) glucose and (J) GDF15 levels before and after 7 days of overfeeding (48 ± 1 % greater than estimated daily requirements) in non-obese healthy human volunteers. Data is from 28 adult participants and expressed as mean ± SEM compared using a paired Two tailed Student t-test, with leptin expressed as median (IQR) compared using a Wilcoxon signed rank test, *$p < 0.05$, **** $p < 0.0001$.

HS6-Human study 6 (K-M)**:** (K) Body weight (L) leptin and (M) GDF15 following four or eight weeks of overfeeding (additional 40 % weight maintenance energy requirements) in 20 volunteers. Data is expressed as mean ± SEM. $p < 0.01$, ****$p < 0.0001$ by a One-way ANOVA with Bonferroni multiple comparison post-test **

Figure S2

MS2

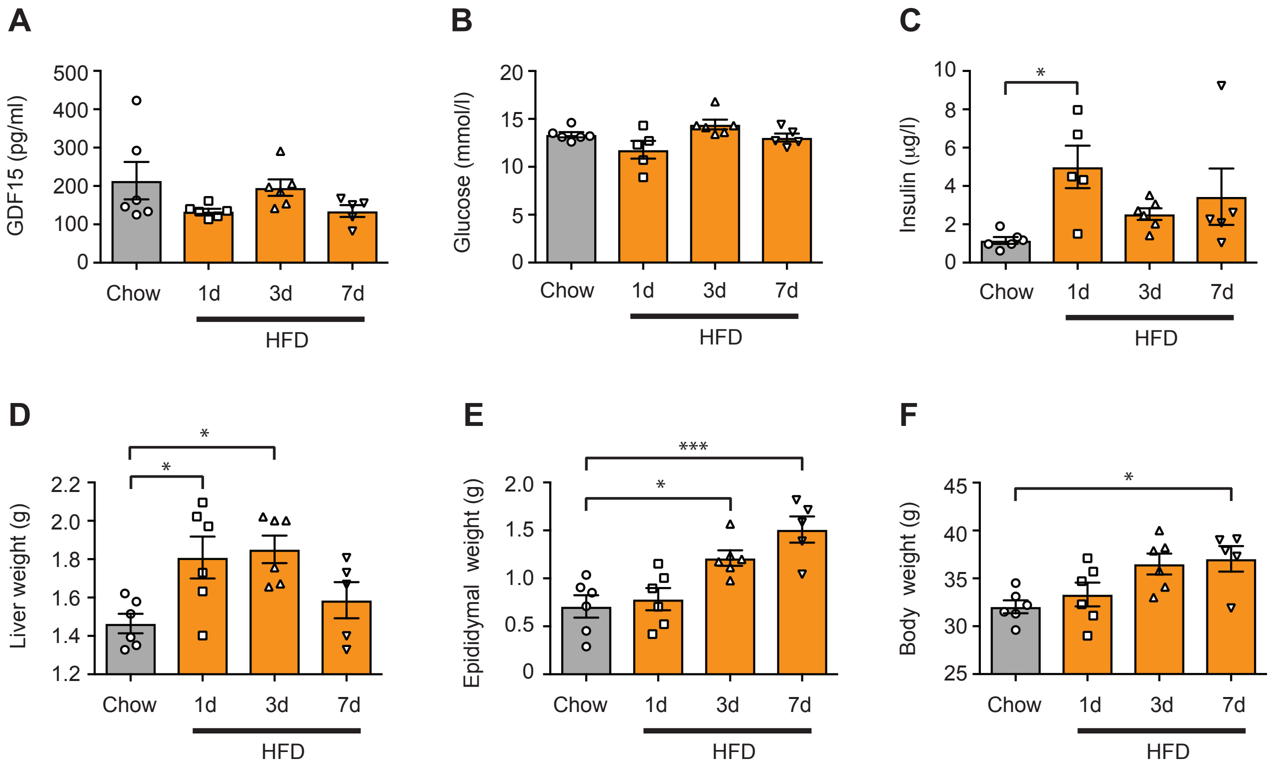

Figure S2 (related to Figure 2): **GDF15 is unaffected by acute HFD (high-fat diet) feeding in mice.**

MS2-Mouse study 2: Plasma (A) GDF15, (B) blood glucose and (C) plasma insulin concentrations from 17-18 week-old male mice that were either fed a chow diet or subjected to a 45% HFD for 1-7 days (d). (D) Liver, (E) epididymal adipose tissue and (F) body weight from each diet group. Data is expressed as mean ± SEM (n=6 mice per group, except 7d, n=5). * $p<0.05$, ***$p < 0.001$ by One way ANOVA with Bonferroni multiple comparison post test.

Figure S3

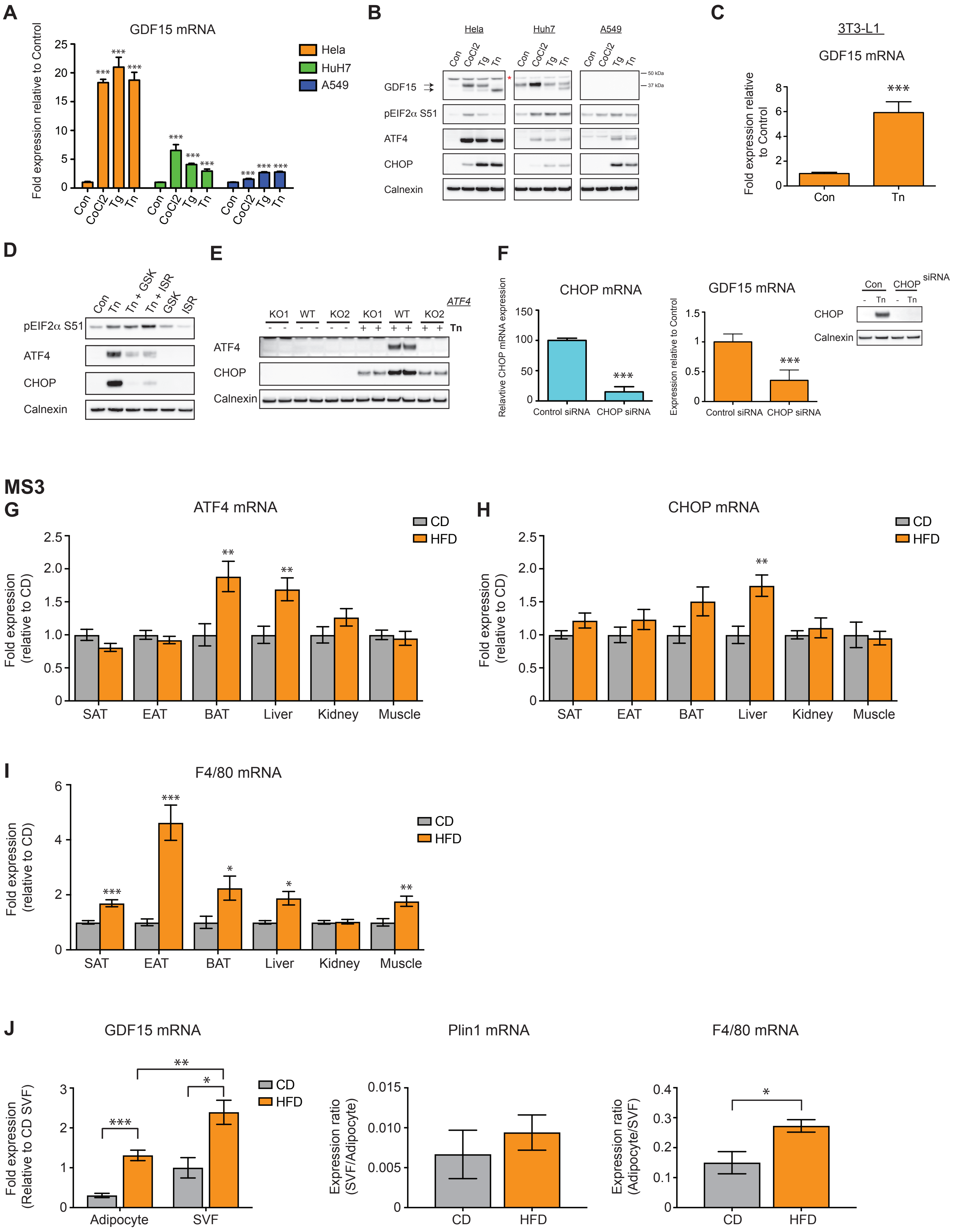
A
GDF15 mRNA
Fold expression relative to Control
Hela
HuH7
A549
Con
CoCl2
Tg
Tn
B
Hela
Huh7
A549
GDF15
pEIF2α S51
ATF4
CHOP
Calnexin
50 kDa
37 kDa
C
3T3-L1
GDF15 mRNA
Fold expression relative to Control
Con
Tn
D
Tn + GSK
Tn + ISR
GSK
ISR
E
ATF4
KO1
WT
KO2
Tn
F
CHOP mRNA
Relavtive CHOP mRNA expression
Control siRNA
CHOP siRNA
GDF15 mRNA
Expression relative to Control
siRNA
MS3
G
ATF4 mRNA
CD
HFD
Fold expression (relative to CD)
SAT
EAT
BAT
Liver
Kidney
Muscle
H
CHOP mRNA
I
F4/80 mRNA
J
GDF15 mRNA
Fold expression (Relative to CD SVF)
Adipocyte
SVF
Plin1 mRNA
Expression ratio (SVF/Adipocyte)
F4/80 mRNA
Expression ratio (Adipocyte/SVF)

Figure S3 (related to Figure 3). GDF15 is regulated by the cellular integrated stress (ISR) response pathway. (A) GDF15 mRNA expression and (B) immunoblot analysis from cell lysates for ISR components in Hela, HuH7 and A549 cells treated with cobalt chloride ($CoCl_2$, 625 uM), thapsigargin (Tg, 1 μM), tunicamycin (Tn 5 μg/ml) or L-Histidinol (His, 1 mM) for 6 h. Note for (B), the arrows denote GDF15 protein and that Tn treatment causes a mobility shift that we hypothesize is due to an impairment of GDF15 glycosylation. Red asterisks indicates a non-specific band. Whilst GDF15 mRNA was induced in (A), there was no detectable GDF15 protein in A549 cells in cell lysates. (C) GDF15 mRNA expression in 3T3-L1 preadipocytes treated with Tn (5 μg/ml) for 6 h. (D) Immunoblot analysis from cell lysates for ISR components in WT MEFs treated with TN in the presence or absence of the PERK inhibitor GSK2606414 (GSK, 200 nM) or eIF2a inhibitor ISRIB (ISR, 100 nM) or (E) in Tn-treated WT or ATF4 KO MEFs. GDF15 mRNA is presented as fold expression relative to its respective control treatment for each cell type (set at 1), normalised to HPRT gene expression in MEFs and 3T3-L1 and GAPDH in human cells. Data is expressed as mean ± SD from three independent experiments. Blots shown are a representative of three independent experiments with Calnexin used as a loading control. (F) CHOP or GDF15 mRNA expression in control siRNA and CHOP siRNA transfected WT MEFs, right hand panel, immunoblot analysis from cell lysates showing the effectiveness of CHOP siRNA on Tn-induced (5 μg/ml – 6 h) CHOP protein expression. mRNA data is mean ± SD from three independent experiments with control treated cells set as 100. ***$p < 0.001$ by Two tailed Student T-Test. Blot shown is a representative of three independent experiments with Calnexin used as a loading control.

GDF15 upregulation in high-fat fed mice is associated with induction of ISR (integrated stress response) pathways. MS3-Mouse Study 3: (A) ATF4 (B) CHOP and (C) F4/80 mRNA expression in subcutaneous - (SAT), epididymal - (EAT) and brown (BAT) adipose tissue, liver, soleus muscle and kidney isolated from C57Bl/6J male mice fed a chow - (CD) or high-fat diet (HFD) for 18 weeks (n = 6-8 mice/group). mRNA is presented as fold expression (mean ± SEM) relative to CD (set at 1) and normalised to the geometric mean of B2M/36b4 gene expression. * $p < 0.05$, ** $p < 0.01$, ***$p < 0.001$ by Two tailed Student T-Test. (D) GDF15, Plin1 and F4/80 mRNA expression in adipocyte and stromo- vascular fractions (SVF) from 18 weeks CD or HFD epididymal adipose tissue. For GDF15, mRNA data is presented as fold-expression relative to chow fed SVF, whereas for Plin1 and F4/80, is presented as ratio between the two fractions, with all data normalised to geometric mean of 36b4/HPRT. Data is expressed as mean ± SEM and analysed by Two way ANOVA with Bonferroni multiple comparison post test for GDF15 and Two tailed Student T-Test for Plin1 and F4/80 * $p < 0.05$, ** $p < 0.01$, ***$p < 0.001$.

Figure S4

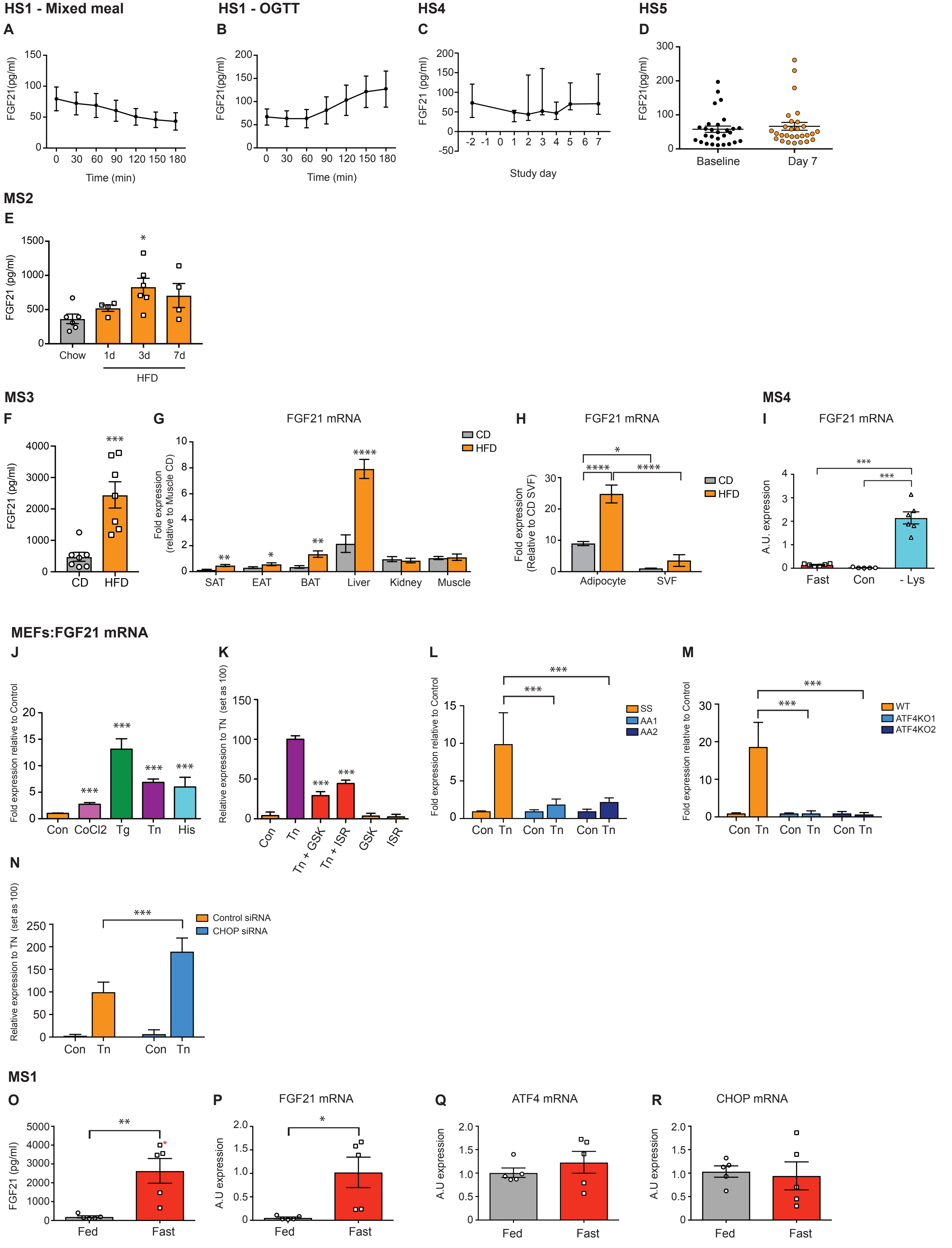
HS1 - Mixed meal
HS1 - OGTT
HS4
HS5
A
B
C
D
FGF21(pg/ml)
FGF21 (pg/ml)
Time (min)
Study day
Baseline
Day 7
MS2
E
Chow
1d
3d
7d
HFD
MS3
F
G
H
FGF21 mRNA
CD
HFD
Fold expression (relative to Muscle CD)
SAT
EAT
BAT
Liver
Kidney
Muscle
Fold expression (Relative to CD SVF)
Adipocyte
SVF
MS4
I
A.U. expression
Fast
Con
- Lys
MEFs:FGF21 mRNA
J
K
L
M
Fold expression relative to Control
Con
CoCl2
Tg
Tn
His
Relative expression to TN (set as 100)
Tn + GSK
Tn + ISR
GSK
ISR
SS
AA1
AA2
WT
ATF4KO1
ATF4KO2
N
Control siRNA
CHOP siRNA
MS1
O
P
Q
R
ATF4 mRNA
CHOP mRNA
A.U expression
Fed
Fast

Figure S4 (related to Figures 1-4). FGF21 regulation in response to nutritional challenges in mice and humans.

HS1-5 – Human Studies 1-5. (A-C) Circulatory levels of FGF21 in volunteers that participated in meal or imposed caloric deficit or (D) overfeeding studies. MS2-4- Mouse Studies 2-4. Plasma FGF21 and tissue mRNA expression in mice subjected to (E) short-term or (F-H) Long-term high fat or (I) lysine deficient diet. (J-N) FGF21 mRNA expression and its regulation by the cellular intergrated stress response pathways in MEFs . MS1- Mouse study 1. (O) Plasma FGF21 and (P-R) hepatic FGF21, ATF4 and CHOP mRNA expression in mice subjected to fasting. The experimental details and the statistical analysis for FGF21 are identical to those conducted for GDF15 (see main and supplemental figures).
